# The global estimate of premature cardiovascular mortality: a systematic review and meta-analysis of age-standardized mortality rate

**DOI:** 10.1186/s12889-023-16466-1

**Published:** 2023-08-16

**Authors:** Wan Shakira Rodzlan Hasani, Nor Asiah Muhamad, Tengku Muhammad Hanis, Nur Hasnah Maamor, Xin Wee Chen, Mohd Azahadi Omar, Yee Cheng Kueh, Zulkarnain Abd Karim, Muhammad Radzi Abu Hassan, Kamarul Imran Musa

**Affiliations:** 1 Department of Community Medicine, School of Medical Sciences, Universiti Sains Malaysia, Kubang Kerian, 16150 Kelantan, Malaysia; 2 Institute for Public Health, National Institutes of Health, Ministry of Health Malaysia, Setia Alam 40170, Selangor, Malaysia; 3 Sector for Evidence-Based Healthcare, National Institutes of Health, Ministry of Health, Shah Alam, Selangor Malaysia; 4 Department of Public Health Medicine, Faculty of Medicine, Universiti Teknologi MARA, Sungai Buloh Campus, 47000 Selangor, Malaysia; 5 Sector for Biostatistics and Data Repository, National Institutes of Health, Ministry of Health Malaysia, Setia Alam 40170, Selangor, Malaysia; 6 Biostatistics and Research Methodology Unit, Kubang Kerian, 16150 Kelantan, Malaysia; 7 Office of The Manager to Biomedical Research Policy & Strategic Planning Unit, Institutes for Medical Research, Setia Alam 40170, Selangor, Malaysia; 8 Office of Director-General, Ministry of Health Malaysia, 62590 Putrajaya, Malaysia

**Keywords:** Premature mortality, Cardiovascular diseases, Age standardized mortality rate

## Abstract

**Background:**

Cardiovascular disease (CVD) is a significant cause of premature mortality worldwide, with a growing burden in recent years. Despite this, there is a lack of comprehensive meta-analyses that quantify the extent of premature CVD mortality. Study addressed this gap by estimating the pooled age-standardized mortality rate (ASMR) of premature CVD mortality.

**Methods:**

We conducted a systematic review of published CVD mortality studies that reported ASMR as an indicator for premature mortality measurement. All English articles published as of October 2022 were searched in four electronic databases: PubMed, Scopus, Web of Science (WoS), and the Cochrane Central Register of Controlled Trials (CENTRAL). We computed pooled estimates of ASMR using random-effects meta-analysis. We assessed heterogeneity from the selected studies using the I^2^ statistic. Subgroup analyses and meta regression analysis was performed based on sex, main CVD types, income country level, study time and age group. The analysis was performed using R software with the “meta” and “metafor” packages.

**Results:**

A total of 15 studies met the inclusion criteria. The estimated global ASMR for premature mortality from total CVD was 96.04 per 100,000 people (95% CI: 67.18, 137.31). Subgroup analysis by specific CVD types revealed a higher ASMR for ischemic heart disease (ASMR = 15.57, 95% CI: 11.27, 21.5) compared to stroke (ASMR = 12.36, 95% CI: 8.09, 18.91). Sex-specific differences were also observed, with higher ASMRs for males (37.50, 95% CI: 23.69, 59.37) than females (15.75, 95% CI: 9.61, 25.81). Middle-income countries had a significantly higher ASMR (90.58, 95% CI: 56.40, 145.48) compared to high-income countries (21.42, 95% CI: 15.63, 29.37). Stratifying by age group indicated that the age groups of 20–64 years and 30–74 years had a higher ASMR than the age group of 0–74 years. Our multivariable meta-regression model suggested significant differences in the adjusted ASMR estimates for all covariates except study time.

**Conclusions:**

This meta-analysis synthesized a comprehensive estimate of the worldwide burden of premature CVD mortality. Our findings underscore the continued burden of premature CVD mortality, particularly in middle-income countries. Addressing this issue requires targeted interventions to mitigate the high risk of premature CVD mortality in these vulnerable populations.

**Supplementary Information:**

The online version contains supplementary material available at 10.1186/s12889-023-16466-1.

## Background

Premature mortality refers to deaths that occur at a younger age than expected, based on the average life expectancy [1] 00 [2]. According to the World Health Organization (WHO), low- and middle-income countries (LMICs) experience a disproportionately high burden of premature mortality compared to high-income countries (HICs) [3]. Even though CVD mortality rates have decreased dramatically over the past two decades, the burden of premature CVD mortality is on the rise in LMICs, emphasizing the need for continued efforts to prevent and manage CVD in these regions [4]. In 2015, the WHO developed an ambitious target by 2030 to reduce by one-third premature mortality from non-communicable diseases (NCDs) through the Sustainable Development Goal (SDG) [5]. The urgency needs to be focused on revising existing policies for the preventing and controlling NCDs, including CVDs [6]. In order to ensure the SDG’s target is on track, information on premature CVD mortality may assist in developing global and context-specific strategies for reducing the incidence of premature CVD mortality.

Age-standardized mortality rate (ASMR) is a commonly used measure to assess premature mortality in a population. ASMR adjusts for differences in the age distribution of populations, which can vary widely between countries or regions [7]. By controlling for these differences, ASMR allows comparisons between populations with different age structures. ASMR can be used to monitor changes in premature mortality over time and to compare mortality rates between different populations. It is a useful tool for identifying health disparities and evaluating the impact of public health interventions. The age limit for calculating ASMR for premature mortality varies depending on the individual context and purpose of the analysis. WHO considers an ASMR for premature mortality between the ages of 30 and 70 years [8], while some studies report an ASMR below 65 [9] and 75 [10]. To calculate ASMR, age-specific mortality rates (i.e., the number of deaths within specific age groups divided by the corresponding population size) are applied to a standard population structure. The standard population structure is usually based on the age distribution of a reference population, such as a national or international standard. The age-specific mortality rates for each age group are then weighted based on the standard population structure, and the weighted rates are summed to obtain the ASMR. The ASMR is then calculated as the ratio of the expected number of deaths to the corresponding standard population size, expressed as a rate per 100,000 or 1,000 population [11].

While numerous systematic reviews and meta-analyses have explored CVD mortality, few have specifically examined premature mortality as an outcome measure. Instead, these analyses have primarily focused on identifying predictors of increased mortality, risk factors for cause-specific mortality, and relative risks associated with CVD [12–15]. The GBD study, which provides the most widely-used estimates of premature mortality globally, has faced limitations in accurately estimating premature CVD mortality due to inadequate or low-quality mortality data in some countries, particularly in impoverished regions [16, 17]. Hence, there is scarcity of a comprehensive systematic review with meta-analysis that estimates the pooled ASMR for premature CVD mortality. To address these gaps, we have undertaken a systematic review and meta-analysis to identify relevant studies and synthesize their findings on ASMR related to premature CVD mortality. Furthermore, we performed a sub-analysis to pool estimates of ASMR by sex, major types of CVD, income country level and time of study. These parameters were selected to enhance our understanding of premature death. Examining sex differences helps uncover sex-related disparities in CVD prevalence and outcomes. Analysing major CVD types allows us to address unique risk factors and design targeted interventions. Assessing premature mortality across income country levels helps us identify socioeconomic disparities and tailor strategies accordingly. Finally, studying premature mortality trends over time enables us to monitor progress and evaluate the effectiveness of public health interventions. By incorporating these parameters, our study aims to provide a comprehensive evaluation of premature CVD mortality and contribute to the development of global strategies to combat this pressing public health issue.

## Methods

### Protocol and registration

We registered the protocol for this review with the International Prospective Register of Systematic Reviews (PROSPERO), [Registration number: CRD42021288415]. We conducted this review in accordance with the Preferred Reporting Items for Systematic Reviews and Meta-analysis (PRISMA) [18]. Detail methodology of this review has been explained elsewhere [19]. For purpose of this review, we will report the pool estimates of ASMR for premature CVD mortality.

### Search strategy

We conducted searches in electronic databases namely PubMed, Scopus, Web of Science (WoS), and the Cochrane Central Register of Controlled Trials (CENTRAL) to identify potential studies. Additionally, we searched Google Scholar to identify articles not found in the major electronic databases. The search was conducted in all databases up to October 18, 2022. Our search strategy included the Mesh term 'cardiovascular diseases' in combination with terms for specific cardiovascular conditions (e.g., coronary heart disease, cerebrovascular disorder, myocardial ischemia, or stroke). We also included a term for premature mortality (e.g., premature death, premature mortality, age standardized mortality rate or years of life lost). The “AND” Boolean operator was used to combine search terms across the categories and the “OR” was used to combine within the categories. We expanded the search by reviewing the reference lists of included articles to identify any additional eligible studies. To focus on relevant primary studies, we excluded review articles from our search strategy. Furthermore, the search was limited to studies published in the English language. The detailed search terms for each database are presented in Supplement 1.

### Inclusion and exclusion criteria

All eligible studies had to satisfy the following criteria: (1) reported premature mortality or death from cardiovascular disease; (2) measure premature mortality using ASMR; (3) defined age limit for premature mortality (e.g., 0–64 years, 0–74 years); (4) reported ASMR per 100,000 population; (5) used primary data or observational data on cause-specific mortality from CVD; (6) follow International classification of diseases (ICD) code for CVD death (e.g., ICD-10 code for CVD: I01-I99); (7) reported number of deaths from CVD, or 95% confident interval (CI) allowing standard error (SE) to be calculated. The studies were excluded if when they; (1) reported mortality in a particular subgroup of the population or specific cohort (e.g., congenital heart disease, epilepsy or pregnant women); (2) used estimated data (e.g., data from GBD study or any global estimated data); (3) reported CVD as a combination with other diseases (e.g., NCD or all cause premature mortality; or (4) reported duplicate data (or data sets from overlapping periods at the same site).

### Screening process

**Fig. 1:**
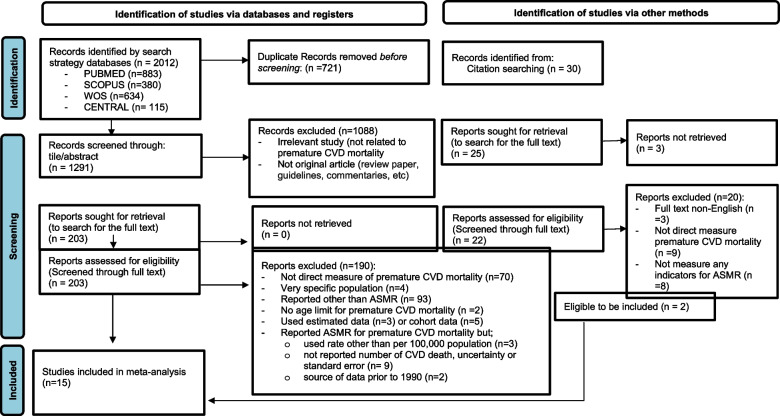
Flow diagram of the published articles evaluated for inclusion in this review

One review author (W.S.R.H) removed all duplicate publication prior to titles and abstracts screening. Two authors (W.S.R.H and C.X.W) independently screened the titles and abstracts to examine the potential studies for inclusion and exclude those that were obviously irrelevant. Another two independent review authors (W.S.R.H and T.M.H) independently screened the full-text for inclusion according to the eligibility criteria, and documented reasons for exclusion for all excluded studies. We resolved any disagreements through discussion. If necessary, a third review author (N.A.M) was consulted to provide input. If consensus could not be reached, another author (K.I.M) acted as an arbiter. All references were stored, organized, and managed using Mendeley Reference Management Software [20]. To ensure transparency, we recorded our selection process and completed a flow diagram (Fig. 1) in accordance with the PRISMA guidelines [18].

### Data extraction and management

Data extraction was performed independently by two review authors (W.S.R.H and T.M.H) following the Cochrane Handbook for Systematic Reviews of Interventions [21]. We utilized a standardized data extraction form developed using a Microsoft Excel spreadsheet to record study characteristics and outcome data. One review author (W.S.R.H) conducted a comprehensive extraction of all data, subsequently verified for accuracy by a second review author (T.M.H). For all eligible articles, we extracted information including the first author's name, year of publication, study design, country, source of data, year of data source, study population, age coverage for premature mortality, number of deaths, types of CVD death, ICD code for cause of death, the method or formula used for ASMR calculation and the value of ASMR per 100,000 population with their respective 95% CI if provided. The ASMR value was categorised separately based on sex, CVD types and study time. We contacted the author to get the exact value for ASMR if they reported it in the plot or if ASMR was reported as part of all-cause or general NCD mortality. For each study included in our analysis, ASMR values could be reported for different subgroups such as each sex, CVD type or multiple years. To ensure comprehensive analysis, we treated each ASMR value as a separate data point extracted from each study. In cases where studies reported multiple years within a given time interval (e.g., each year from 2010–2019), we selected ASMR values from the earliest, middle, and latest years (e.g., 2010, 2015, and 2019) to represent the ASMR for the entire period, provided there was no significant variation across those years. However, if a study reported a single ASMR value for the entire year range (e.g., 1999–2018), we used that value as the estimate for the entire year. It is important to note that data from some countries might have limited quality and representativeness. To address this, we made two assumptions in our analysis: a) the data from each source represents the national population, and b) data measurement was valid for all data sources.

### Quality assessment

The quality of the included studies was assessed through an adapted version of the Newcastle–Ottawa Scale (NOS) for cross-sectional studies and modified this version to better suit the specific characteristics of our study [22]. The NOS is a widely used tool for assessing the quality of non-randomized studies, such as observational and case–control studies [23]. The adopted version of NOS assesses the same three components (selection, comparability, and outcome) as the original version. The score for the adapted version of the cross-sectional studies is as follows: 1) very good studies: 9–10 points; 2) good studies: 7–8 points; 3) satisfactory studies: 5–6 points; and 4) unsatisfactory studies: 0–4 points. The detailed criteria for NOS assessment are represented in Supplement 2. Two review authors (W.S.R.H and N.H.M) assessed the quality of each study using our adapted NOS tool and resolved any discrepancies through discussion or by consulting a third reviewer (N.A.M). We reported the details results of the quality assessment in a separate table (presented in Supplement 2), which included the total score for each study and the scores for each item. We also presented the overall quality score for each study in Table 1, along with the characteristics of the included studies.

**Table 1:** Characteristics of included studies

No	Author (Year)	Study time	Study design	Country	Population	Age range	Data source	CVD type	ICD code	Measured ASMR by sex	Standard population for age adjusted	Quality assessment (NOS)			
												S	C	O	Total score
1	Dani et al*.* (2022) [24]	1999–2019	CS	USA	General	0–64	Vital registration (CDC WONDER database)	AMI	ICD-10 (121–122)	Yes (M, F)	2000 US standard population	⋆⋆⋆⋆	⋆⋆	⋆⋆⋆	9 Very Good
2	Milicevic et al*.* (2009) [25]	2000	CS	Serbia	General	0–69	Vital registration	All CVD	ICD-10 (I00-I99)	Yes (M, F)	Standard population of Europe	⋆⋆⋆⋆	⋆⋆	⋆⋆⋆	9 Very Good
3	Yang et al*.* (2021) [26]	2007–2018	CS	China	General	30–69	Vital registration	All CVD	ICD-10 (I00-I99)	No	2000 China’s Fifth Census Data	⋆⋆⋆⋆	⋆	⋆⋆⋆	8 Good
4	Istilli et al*.* (2020) [27]	2010–2014	CS	Brazil	General	30–69	Vital registration	All CVD	ICD-10 (I00-I99)	Yes (M, F)	WHO standard population	⋆⋆⋆⋆⋆	⋆⋆	⋆⋆⋆	10 Very Good
5	Puska et al. (1998) [28]	1993–1995	CS	Finland	General	35–64	Vital registration	All CVD, IHD, CBVD	ICD-9 (350–459) ICD-9 (410–414) ICD-9 (430–438)	Yes (M, F)	World Standard Population	⋆⋆⋆⋆	⋆⋆	⋆⋆⋆	9 Very Good
6	Moryson & Stawińska (2022) [29]	2008–2017	CS	Poland	General	20–64	Vital registration	All CVD, IHD, CBVD	ICD-10 (I00-I99), ICD-10 (I20-I25), ICD-10 (I60-I69)	Yes (M, F)	2013 Standard European population	⋆⋆⋆⋆⋆	⋆⋆	⋆⋆⋆	10 Very Good
7	Hervella et al*.* (2021) [30]	1998–2018	CS	Spain	General	0–74	Vital registration	IHD	ICD-10 (I20-I25), ICD-9 (410–414)	Yes (M, F)	2013 European standard population	⋆⋆⋆⋆⋆	⋆⋆	⋆⋆⋆	10 Very Good
8	Best et al*.* (2018) [31]	1990; 2016	CS	USA	General	25–64	Vital registration (CDC database)	Heart disease	ICD-10 (I00-I09, I11, I13, I20-I51)	Yes (M, F)	2000 US population	⋆⋆⋆⋆	⋆⋆	⋆⋆⋆	9 Very Good
9	Wijnen et al*.* (2022) [32]	2006–2016	CS	Australia	General	30–69	Vital registration	All CVD	ICD-10 (I00-I99)	No	2011 Resident Population of Australia	⋆⋆⋆⋆⋆	⋆	⋆⋆⋆	9 Very Good
10	Pinlac & Soonthornworasiri (2016) [33]	2006–2016	CS	Philipines	General	30–69	Vital registration	All CVD	ICD-10 (I00-I99)	No	Not reported	⋆⋆⋆		⋆⋆⋆	5 Satisfactory
11	Mariani et al*.* (2016) [34]	2000–2011	CS	Argentina	General	0–74	Vital registration	Stroke	ICD-10 (I60-I69)	No	2010 Argentine population	⋆⋆⋆⋆	⋆⋆	⋆⋆⋆	10 Very Good
12	Gómez-Martínez et al*.* (2018) [35]	1999–2013	CS	Spain	General	0–74	Vital registration	Heart failure	ICD-10 (I50)	Yes (M, F)	2013 European standard population	⋆⋆⋆⋆⋆	⋆⋆	⋆⋆⋆	10 Very Good
13	Song et al*.* (2021) [36]	1999–2018	CS	USA	General	25–64	Vital registration (CDC WONDER database)	Stroke	ICD-10 (I60-I69)	Yes (M, F)	2000 US population	⋆⋆⋆⋆⋆	⋆⋆	⋆⋆⋆	10 Very Good
14	Gawryszewski & Souza (2014) [37]	2000–2008	CS	USA	General	30–69	Vital registration (PAHO data)	All CVD	ICD-10 (I00-I99)	Yes (M, F)	WHO Standard Population	⋆⋆⋆⋆	⋆⋆	⋆⋆⋆	8 Very Good
15	Jin et al*.* (2020) [38]	1999–2017	CS	USA	General	35–74	Vital registration (CDC database)	Cardiac disease	ICD-10 (I21, I25, I40, I34, I35, I42, I45-I49, R96, Q20-Q24, Q87)	No	2000 projected US population	⋆⋆⋆⋆⋆	⋆⋆	⋆⋆⋆	10 Very Good

^*^Note: *AMI* Acute myocardial infarction, *CBVD* cerebrovascular disease or stroke, *CDC* Center for Disease Control, *CS* Cross-sectional study design, *CVD* cardiovascular disease, *ICD-9 code* International Classification of Diseases 9^th^ Revision code, *ICD-10 code* International Classification of Diseases 10^th^ Revision code, *IHD* Ischemic heart disease, *F* Female, *M* Male, *NOS* Newcastle–Ottawa Scale (S: selection, C: comparability, and O: outcome). *PAHO* Pan-American Health Organization. Gawryszewski and Souza (2014) utilized PAHO data from 27 countries across the Americas, including North America, Latin America, and the non-Latin Caribbean region. Study time: time for the data source. Vital registration, including the national death registry, mortality database, or censuses. WONDER: Wide-Ranging Online Data for Epidemiologic Research

### Statistical analysis

We manually calculated the SE for each study's ASMR estimate before running the meta-analysis. For studies that reported a 95% CI, we used the formula SE = (upper limit of CI—lower limit of CI) / 3.92 [21]. For studies that reported the number of deaths, we used the formula SE = R/ square root of N, where R is age adjusted rate and N is numbers of death [39]. These manual calculations allowed us to standardize the SEs across studies and incorporate them into the meta-analysis using a random-effects model. We verified our manual calculations by comparing them to the SEs reported in the studies' original publications, and found them to be consistent.

To estimate the pooled ASMR from CVD across multiple studies, we used the “meta” package [40] and “metafor” [41] package in R. We first imported the data from the included studies into R and performed any necessary data cleaning and processing. We then used the *metagen* function to fit a random-effects model to the ASMR estimates. In our meta-analysis, we chose a random-effects model due to the observed heterogeneity among the included studies. This model accounts for both within-study and between-study variation in the effect sizes, providing a more conservative estimate of the pooled effect size. We specified the effect size measure as the log-transformed ASMR. We also used the *forest* function to generate a forest plot of the individual study effect sizes and their 95% CI, including a horizontal line representing the overall effect estimate. In addition, we estimated a prediction interval (PI) for the overall effect size estimate. The calculation of the PI takes into account the between-study variance (estimated tau-square) in addition to the within-study variance, providing a more comprehensive range of plausible effects. This is shown as a horizontal line on the forest plot, extending beyond the limits of the CI and indicating the plausible range of effects [42]. To investigate potential sources of heterogeneity, we initially classified the studies based on by sex (male or female), CVD type (total CVD, IHD, stroke, or other), study time (1990–1999, 2000–2009 or after 2010) and income country level; HICs or middle-income countries, MICs (which included low and upper middle-income countries). Subsequently, we fitted separate random-effects models for each subgroup, allowing for a more focused analysis within each category.

We also conducted a multivariable meta-regression analysis to investigate the associations between various parameters. The analysis was performed using the 'rma' function in the 'metafor' package in R [41]. During the classification of studies into three distinct time periods (1990–1999, 2000–2009, and after 2010), we excluded two studies. These studies reported a single ASMR value derived from mortality data spanning the years 2000 to 2019. The backward selection and likelihood ratio test was used for the covariate selection and model comparison, respectively. Firstly, all covariates were included in the meta-regression model (full model). A covariate with the highest p-value was excluded one at a time (reduced model). Then, the full model was compared to the reduced model using a likelihood test (p value < 0.05) and lower AIC with a correction for small sample size (AICc) indicates the model was better, thus it was selected. Additionally, a model with lower AICc would be selected even if the p value > 0.05. This process was repeated until all covariates had p value < 0.05. Then, the final model was tested for all possible two-way interactions. Finally, the permutation test was done to ensure the robustness of the model as recommended by several studies [43, 44].

### Assessment of heterogeneity

In assessing heterogeneity, we employed various methods. Firstly, the I-squared (I^2^) statistic was used to quantify the proportion of total variation in effect sizes attributable to heterogeneity beyond chance. To categorize the level of heterogeneity, we considered I^2^ values of 25%, 50%, and 75% as representing low, moderate, and high heterogeneity, respectively [45]. To further examine heterogeneity, we visually inspected the forest plots, which displayed the effect sizes of individual studies along with their corresponding CI. This allowed us to identify any outliers or clusters of effect sizes that might contribute to heterogeneity. Formally testing for heterogeneity was accomplished using Cochran's Q test, which assumes a null hypothesis of homogeneity. A p-value of less than 0.01 was considered indicative of significant heterogeneity [46]. In addition to the I^2^ statistic and the Cochran's Q test, we also used the tau (*τ*) statistic to quantify the amount of heterogeneity in the meta-analysis. This statistic takes into account sampling error and true heterogeneity and provides a measure of between-study variance. A larger tau value indicates greater heterogeneity across studies. To explore potential sources of heterogeneity, we conducted subgroup analyses based on various factors, including sex, CVD type, income country level, and study time.

### Assessment of reporting biases

To evaluate potential reporting biases in our meta-analysis, we employed several approaches. Firstly, we utilized a funnel plot, which plots the effect size estimate (ASMR) of each study on the x-axis and its corresponding standard error or precision on the y-axis. This plot enables the detection of asymmetry, which could suggest the presence of smaller studies with larger effect sizes missing from the lower left corner. However, it is important to consider that asymmetry in a funnel plot may indicate publication bias, but other factors like heterogeneity or chance can also contribute to the asymmetry [47]. Apart from the funnel plot, we used Begg's [48] and Egger's tests [49] to further assess reporting bias in our meta-analysis. Begg's test is a rank correlation test that examines the association between the effect size and its variance across studies [48]. A significant p-value indicates potential presence of publication bias or small-study effects, where smaller studies with larger effect sizes are more likely to be published. On the other hand, Egger's test is a regression-based test that examines the asymmetry in the funnel plot by analysing the relationship between the effect size and its standard error [49]. A significant p-value from this test suggests the potential presence of publication bias or small-study effects.

### Sensitivity analysis

In order to assess the robustness of our findings, we conducted a sensitivity analysis, which involved several steps. First, we utilized the Baujat plot, introduced by Baujat et al. (2002) [50] as a diagnostic tool to identify outliers within our meta-analytic data. This plot helps visualize the relationship between the overall result and the contribution of overall heterogeneity for each study included in the meta-analysis. By examining this plot, we were able to identify potential outliers, which are studies that exert a substantial influence on the overall results and may contribute to heterogeneity. Following our criteria for outlier exclusion, we proceeded to re-analysed the data both with and without the identified outlier studies. This allowed us to assess the impact of these outliers on the overall effect estimate, confidence intervals, and heterogeneity. By comparing the results from these two analyses, we could evaluate the robustness of our conclusions and determine whether the outliers significantly influenced our findings. Furthermore, we recognized that the differences in age thresholds used to define premature mortality could potentially introduce heterogeneity and bias into our results. To address this concern, we conducted additional sensitivity analyses based on different age thresholds. Specifically, we divided the age group into three predefined categories for premature mortality: i) 0–74 years old, ii) 30–74 years old, and iii) 20–64 years old. By examining the impact of these different age thresholds on the overall findings, we aimed to explore their potential influence on the conclusions of our study.

## Results

### Study characteristics

Fifteen out of 2012 records identified by search strategy databases and 30 records identified from citation searching were included in the meta-analysis (Fig. 1). They were published between 1998 and 2022, had cross-sectional study design, and used data source from the national vital registration systems covering a period from 1990 to 2019, which represent the general population of samples. These studies were from diverse geographic regions around the globe. Additionally, all studies reported cause of death using ICD codes, ensuring consistency in the definition of outcomes across studies. Eight studies reported CVD as total CVD, and some [24, 28–31, 34–36, 38] reported CVD as a specific CVD type (IHD, stroke, or others). Moreover, ten studies reported ASMR for premature CVD mortality by sex. Quality assessment using the NOS adopted version showed none of the included studies as poor quality (13 were rated as very good, one as good, and one as satisfactory), hence all 15 studies were included (Table 1).

### Overall ASMR estimates and subgroup analysis

**Fig. 2:**
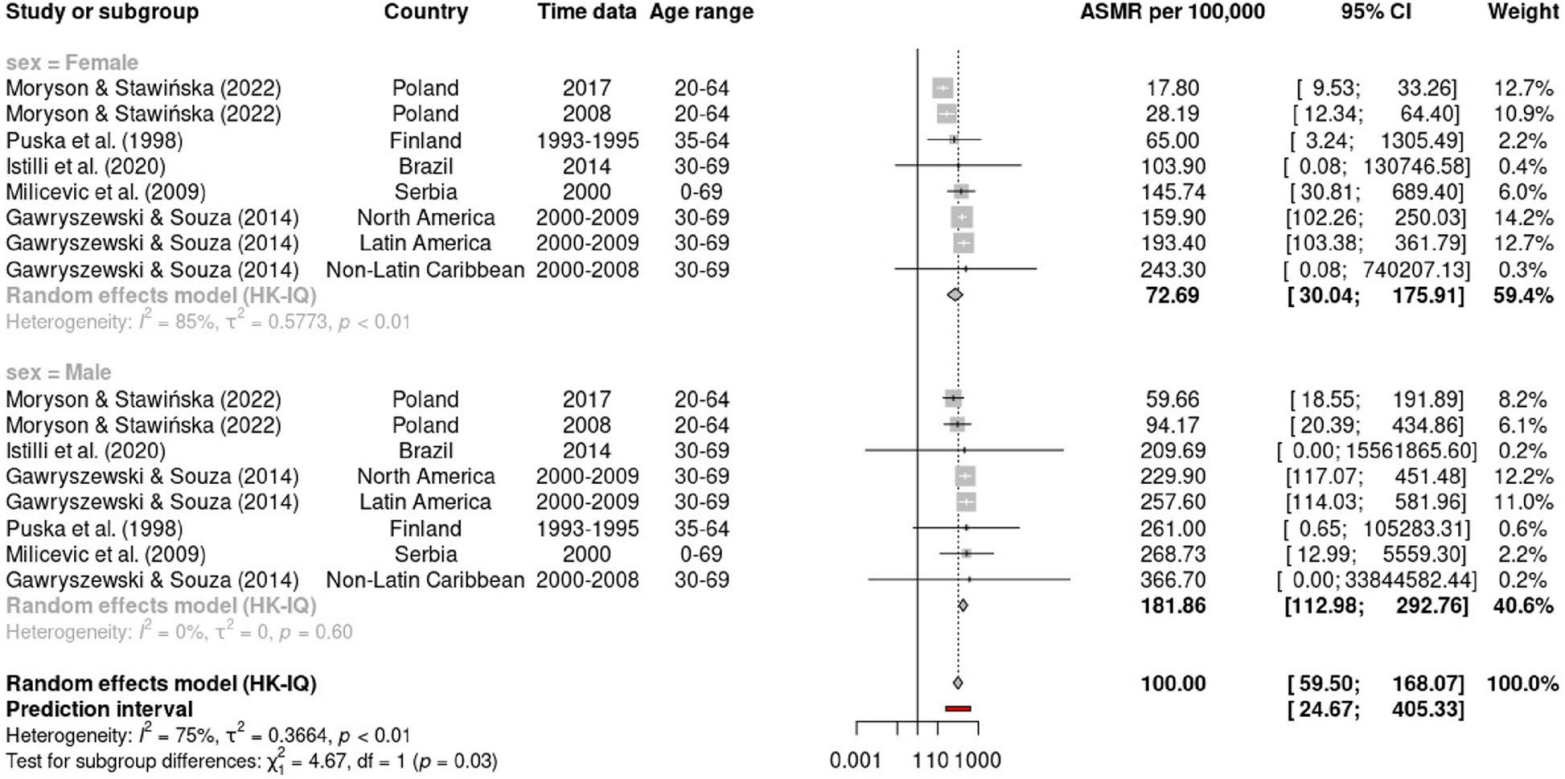
Forest plot sex-specific premature mortality (ASMR per 100,000 population) from all CVD (ICD-10 codes I00-I99 or ICD-9 codes 350–459)

**Fig. 3:**
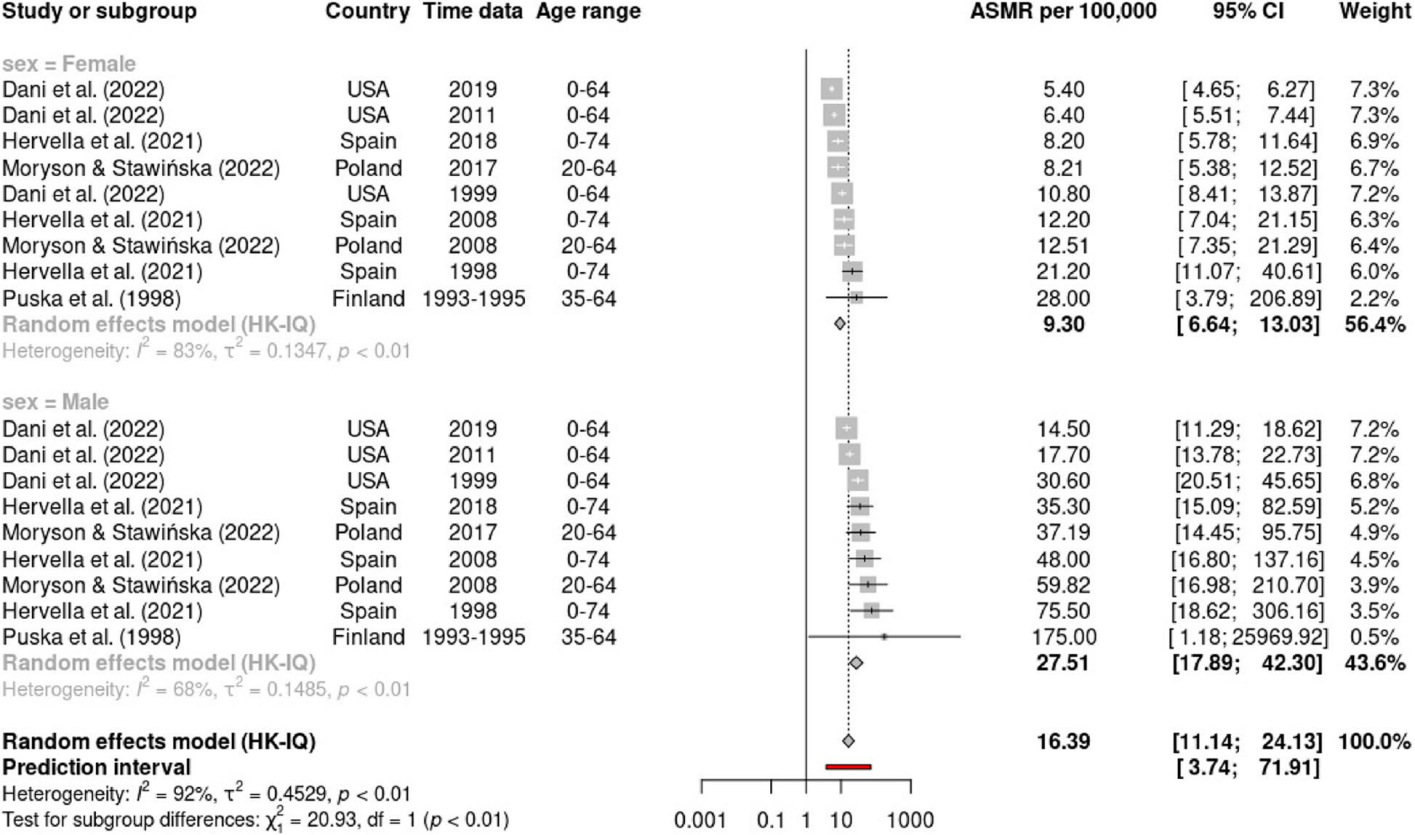
Forest plot sex-specific premature mortality (ASMR per 100,000 population) from IHD (ICD-10 codes I20-I25 or ICD-9 codes 410–414)

**Fig. 4:**
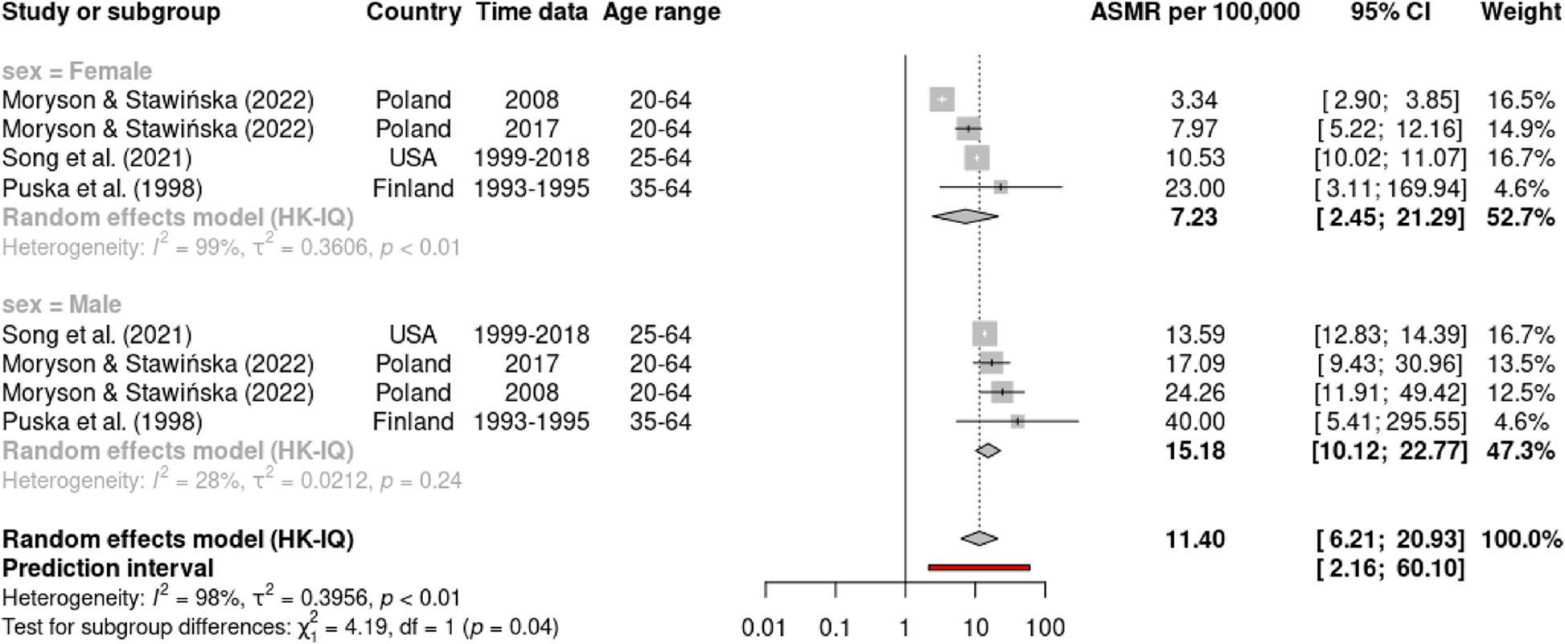
Forest plot sex-specific premature mortality (ASMR per 100,000 population) from cerebrovascular disease or stroke (ICD-10 codes I60-I69 or ICD-9 codes 430–438)

Using a random effects model, Table 2 presents the summary of the pooled estimate of ASMR for premature CVD mortality, in overall and based on the subgroup by CVD types, country income level, and time of study. The meta-analysis estimated the overall ASMR for premature CVD mortality to be 27.0 (95% CI: 20.13, 36.21) per 100,000 people, with a high degree of heterogeneity among the studies (I^2^ = 99%). The subgroup analysis for different types of CVD, shows the ASMR for total CVD being the highest (ASMR = 96.04, 95% CI: 67.18, 137.31; I^2^ = 84%), followed by IHD (ASMR = 15.57, 95% CI: 11.27, 21.5; I^2^ = 92%) and stroke (ASMR = 12.36, 95% CI: 8.09, 18.91; I^2^ = 97%). We also observed that males demonstrated a higher rate (ASMR = 37.50, 95% CI: 23.69, 59.37; I^2^ = 96%) than females (ASMR = 15.75, 95% CI: 9.61, 25.81; I^2^ = 99%). The forest plot for subgroup analysis for overall studies by CVD types, and sex was presented in the Supplement 3 (Figures S1 and S2). On top of that, the statistically significant differences in ASMRs between sex (*p* < 0.05) were demonstrated in subgroup analysis by CVD types (Figs. 2, 3 and 4), in which ASMR for IHD among males (27.51, 95% CI: 17.89, 42.30) was higher than among females (9.30, 95% CI: 6.64, 13.03) (Fig. 3); and ASMR for stroke among males (15.18, 95% CI: 10.12, 22.77) was higher than among females (7.23, 95% CI: 2.45, 21.29) (Fig. 4).

**Table 2:** Summary of pooled ASMR per 100,000 population for premature cardiovascular mortality

Subgroup	Studies (n)	Random effect model ASMR per 100,000	95% Confidence interval	Test for Heterogeneity		
				I^2^ (%)	T^2^	*p* (Q test)
Overall study	15	27.00	20.13, 36.21	99.00	1.21	0
CVD types						
Total CVD^a^	8	96.04	67.18, 137.31	84.00	0.33	< 0.01
IHD^b^	4	15.57	11.27, 21.51	92.00	0.39	< 0.01
Stroke^c^	4	12.36	8.09, 18.91	97.00	0.29	< 0.01
Other types^d^	3	19.79	8.41, 46.58	100.00	2.11	0
Sex						
Male	10	37.50	23.69, 59.37	96.00	0.97	< 0.01
Female	10	15.75	9.61, 25.81	99.00	1.32	0
Country income level^e^						
HICs	10	21.42	15.63, 29.37	99.00	1.16	0
MICs	6	90.58	56.40, 145.48	77.00	0.31	< 0.01
Time study (year)						
1990–1999	4	16.35	8.35, 32.02	96.00	0.77	< 0.01
2000–2009	5	63.84	35.34, 115.31	98.00	1.27	< 0.01
2010–2019	10	19.93	13.56, 29.30	99.00	0.97	0

*ASMR* age-standardized mortality rate, *I*^*2*^ I statistics, *T*^*2*^ Tau statistics, *Q test* Cochran's Q test, *p p*-value

^a^Total CVD death was based on ICD -10 code: I00-I99 or ICD-9 codes: 350–459

^b^Ischemic heart disease (IHD) death based on ICD-10 (I20-I25) or ICD-9 (410–414)

^c^Cerebrovascular disease or stroke death based on ICD-10 (I60-I69) or ICD-9 (430–438)

^d^Other types of CVD death including heart disease (ICD-10: I00-I09, I11, I13, I20- I51), heart failure (ICD-10: I50) and cardiac death ICD-10 (I21, I25, I40, I34, I35, I42, I45-I49, R96, Q20-Q24, Q87)

^e^According to World Bank’s classification. HICs = high income countries, MICs = middle-income countries (including upper middle-income countries and low middle-income countries)

Table 2 revealed a significantly higher overall ASMR for premature CVD mortality in MICs (ASMR = 90.58, 95% CI: 56.40, 145.48; I^2^ = 77%) compared to HICs (ASMR = 21.42, 95% CI: 15.63, 29.37; I^2^ = 99%) (see forest plot Figure S3 in Supplement 3). In addition, similar findings were seen when analysing selected studies that reported total CVD (ICD-10 codes I00-I99 or ICD-9 codes 350–459) by country income level, where a higher ASMR estimate was shown in MICs compared to HICs (ASMR = 111.11 vs. 78.21 respectively) (Forest plot Figure S4, in Supplement 3).

ASMR for premature CVD mortality was 16.35 (95% CI: 8.35, 32.02; I^2^ = 96%) for the 1990–1999 period, increased to 63.84 (95% CI: 35.34, 115.31; I^2^ = 98%) for the 2000–2009 period, and then declined to 19.93 (95% CI: 13.56, 29.30; I^2^ = 99%) for the 2010–2019 period (Table 2 and Forest plot in Supplement 3, Figure S5). Similar undulated pattern was revealed in a subgroup analysis of premature CVD mortality rate by income country level based on the study time revealing an increase from 1990–1999 to 2000–2009, followed by a decrease from 2000–2009 to 2010–2019 (Table 3 and Forest plots in Supplement 3, Figures S6 and S7). It should be noted that MICs data during the period 1990–1999 were not available in any of the studies included in our meta-analysis.

**Table 3:** Sensitivity analysis of pooled ASMR per 100,000 population from premature CVD mortality by excluding the studies outlier

	After removed outlier			Comparison with original data		
Subgroup	n	ASMR (95% CI)	I^2^ (%)	n	ASMR (95% CI)	I^2^ (%)
Overall study	13	31.20 (23.81, 40.89)	97	15	27.00 (20.13, 36.21)	99
CVD types						
Total CVD^a^	6	46.69 (28.63, 76.15)	29	8	96.04 (67.18, 137.31)	84
IHD^b^	3	20.67 (12.41, 34.45)	74	4	15.57 (11.27, 21.51)	92
Stroke^c^	3	12.23 (10.55, 14.17)	86	4	12.36 (8.09, 18.91)	97
Sex						
Male	8	46.23 (31.95, 66.90)	93	10	37.50 (23.69, 59.37)	96
Female	8	18.87 (11.99, 29.70)	97	10	15.75 (9.61, 25.81)	99
Country income classification^e^						
HIC	9	26.53 (19.61, 35.91)	97	10	21.42 (15.63, 29.37)	99
MIC	5	121.56 (70.05, 210.95)	40	6	90.58 (56.40, 145.48)	77
Time study (year)						
1990–1999	3	21.40 (14.68, 31.19)	68	4	16.35 (8.35, 32.02)	96
2000–2009	4	112.85 (64.87, 196.32)	85	5	63.84 (35.34, 115.31)	98
2010–2019	9	23.25 (16.76, 32.24)	97	10	19.93 (13.56, 29.30)	99
Specific subgroup analysis						
HIC by study time						
1990–1999	3	21.40 (14.68, 31.19)	68	4	16.35 (8.35, 32.2)	96
2000–2009	3	40.47 (16.19, 101.22)	97	3	40.47 (16.19, 101.22)	99
2010–2019	5	21.84 (14.99, 31.81)	96	6	18.47 (11.89, 28.67)	99
MIC by study time						
2000–2009	3	172.55 (109.64, 271.55)	12	3	172.55 (109.64, 271.55)	12
2010–2019	3	22.10 (3.29, 148.74)	0	4	50.89 (45.62, 56.77)	0
Total CVD^a^ by sex						
Male	4	81.68 (23.63, 282.35)	0	5	181.86 (112.98, 292.76)	0
Female	4	32.36 (11.30, 92.68)	40	5	72.69 (30.04, 175.91)	85
IHD^b^ by sex						
Male	3	45.07 (24.44, 82.13)	0	4	27.51 (17.89, 42.30)	68
Female	3	11.18 (7.42, 16.86)	46	4	9.30 (6.64, 13.03)	83
Stroke^c^ by sex						
Male	2	14.40 (0.65, 316.68)	11	3	15.18 (10.12, 22.77)	28
Female	2	10.54 (8.22, 13.50)	0	3	7.23 (2.45, 21.29)	99

*n* number of studies, *CVD* cardiovascular disease, *ASMR* age standardized mortality rate (ASMR) per 100,000. Random effect model applied pool estimate of ASMR, 95% CI = 95% confident interval of estimated ASMR

^a^total CVD death was based on ICD -10 code: I00-I99 or ICD-9 codes: 350–459

^b^Ischemic heart disease (IHD) death based on ICD-10 (I20-I25) or ICD-9 (410–414)

^c^Cerebrovascular disease or stroke death based on ICD-10 (I60-I69) or ICD-9 (430–438)

^d^Other types of CVD death including heart disease (ICD-10: I00-I09, I11, I13, I20- I51), heart failure (ICD-10: I50) and cardiac death ICD-10 (I21, I25, I40, I34, I35, I42, I45-I49, R96, Q20-Q24, Q87)

^e^According to World Bank’s classification. HICs = high income countries, MICs = middle-income counties (including upper middle-income countries and low middle-income countries)

Outlier removed for each subgroup as below;

– Overall study: Jin et.al, (2020) and Gómez-Martínez et al. (2018)

– Total CVD: Yang et al. (2021) and Gawryszewski & Souza (2014)

– IHD: Dani et al. (2022)

– Stroke: Moryson & Stawińska (2022)

– Male and female: Best et al. (2018) and Gómez-Martínez et al. (2018)

– Study time year 1990–1999: Gómez-Martínez et al. (2018)

– Study time year 2000–2009: Moryson & Stawińska (2022)

– Study time year 2010–2019: Gómez-Martínez et al. (2018)

– High-income countries: Gómez-Martínez et al. (2018)

– Middle-income countries: Yang et al. (2021)

### Sensitivity analysis

Based on the criteria for outlier exclusion as suggested by the Baujat plot, we reanalysed the data with and without these outlier studies to assess the impact on the overall effect estimate, CI, and heterogeneity. The sensitivity analysis revealed an improvement in I^2^ for each subgroup after removing the outlier studies, indicating a reduction in heterogeneity and improved precision of the effect estimates (Table 3). Specifically, for total CVD (I^2^ decreased from 84 to 29%) where the estimated for ASMR reduced to 46.69 per 100, 000. Removing outliers also resulted in a significant decrease in heterogeneity for other subgroup analysis such as MICs improved the I^2^ from 77 to 40% and specific subgroups analysis (such as female stroke), reducing from 99 to 0%. However, it is essential to note that despite the improvement in I^2^ after removing the outlier studies, the pattern and direction of the findings remained consistent with the results obtained before outlier removal. For instance, a higher ASMR among males than females, a higher ASMR in MICs than HICs, and the undulated trend observed with increases and decreases in ASMR from 1990–2019. These results suggest that excluding outlier studies improved the precision of the effect estimates and reduced heterogeneity, it did not substantially alter the overall conclusions of the analysis.

Table 4 presents the estimated ASMR per 100,000 population by three difference age thresholds to defined premature mortality and subgroup according to CVD types, sex, country income classification, and study time for each age group. The sensitivity analysis revealed an improvement in I^2^ particularly for the age group of 30–74 years, with a decrease from 99 to 84%. In this age group, the estimated ASMR for total CVD slightly increased from 96.04 to 119.78 per 100,000 population. This improvement was observed because the majority of the selected studies using this age threshold reported premature mortality as total CVD deaths. The estimated ASMR for the other age groups (0–74 and 20–64 years) also showed slight changes in each subgroup analysis, with minimal changes in I^2^. Despite the variations in the estimated ASMR for each age group compared to the original overall selection of 15 studies, the patterns remained consistent. For example, the ASMR for premature CVD mortality among males remained higher than among females, and the estimated ASMR for MICs remained higher than HICs.

**Table 4:** Sensitivity analysis of pooled ASMR per 100,000 population from premature CVD mortality by difference age threshold

	Aged 0–74 years			Aged 20–64 years			Aged 30–74 years		
Subgroup	n	ASMR (95% CI)	I^2^ (%)	n	ASMR (95% CI)	I^2^ (%)	n	ASMR (95% CI)	I^2^ (%)
Overall study	5	14.37 (8.66, 23.81)	98	3	23.67 (15.96, 35.09)	97	7	101.23 (72.85, 140.67)	84
CVD types									
Total CVD^a^	1	-	-	1	-	-	6	119.79 (84.17, 170.49)	86
IHD^b^	2	14.74 (10.13, 21.44)	94	1	-	-	1	-	-
Stroke^c^	1	-	-	2	10.54 (5.91, 18.80)	98	1	-	-
Sex									
Male	4	19.23 (7.33, 50.48)	97	3	41.83 (25.70, 68.09)	95	3	214.99 (115.47, 400.28)	0
Female	4	8.12 (3.12, 21.17)	99	3	14.87 (8.63, 25.63)	98	3	132.24 (68.74, 254.40)	16
Country income class.^d^									
HIC	3	10.44 (6.42, 16.97)	99	3	23.67 (15.96, 35.09)	97	4	97.91 (60.63, 158.09)	85
MIC	1	-	-	-	-	-	4	106.35 (61.86, 182.84)	84
Time study (year)									
1990–1999	3	13.2 (4.70, 37.12)	98	1	-	-	1	-	-
2000–2009	3	48.09 (16.58, 139.48)	73	1	-	-	1	-	-
2010–2019	4	8.32 (4.48, 15.47)	99	2	29.96 (18.45, 48.65)	92	4	51.26 (44.94, 58.46)	0

*n* number of studies, *CVD* cardiovascular disease, *ASMR* age standardized mortality rate (ASMR) per 100,000. Random effect model applied pool estimate of ASMR, 95% CI = 95% confident interval of estimated ASMR

^a^total CVD death was based on ICD -10 code: I00-I99 or ICD-9 codes: 350–459

^b^Ischemic heart disease (IHD) death based on ICD-10 (I20-I25) or ICD-9 (410–414)

^c^Cerebrovascular disease or stroke death based on ICD-10 (I60-I69) or ICD-9 (430–438)

^d^Income country classification: According to World Bank’s classification. HICs = high income countries, MICs = middle-income counties (including upper middle-income countries and low middle-income countries)

### Meta-regression model

The results of the multivariable meta-regression model, presented in Table 5, aim to investigate the effects of various covariates on the ASMR estimates. The model considered covariates such as CVD types, sex, country income classification, study time, and age group. Comparing the models, the reduced model (excluding study time) demonstrated a better fit (AICc = 161.38) than the full model (AICc = 166.72), indicating a more accurate representation of the data. To validate the robustness of the final model, a permutation test was performed, confirming that all covariates retained their statistical significance. This strengthens the reliability of the observed associations. In summary, the meta-regression model revealed that IHD, other heart disease, and total CVD had higher adjusted ASMR estimates compared to stroke as the reference category, with coefficient estimates of 1.55, 0.92, and 1.67, respectively. Males had higher ASMR estimates (β = 1.01) compared to females, and MICs (β = 1.20) exhibited higher ASMR estimates compared to HICs. Age groups of 20–64 years (β = 1.41) and 20–74 years (β = 2.14) showed significantly higher ASMR estimates compared to the age group of 0–74 years, highlighting notable differences in mortality rates across different age ranges to define premature CVD mortality.

**Table 5:** Multivariable meta-regression model of ASMR estimates from premature CVD mortality

Covariates	Adjusted β (95% CI)	t value	*P* value
CVD types			
Stroke	Ref.		
IHD	1.55 (0.73, 2.37)	3.80	< 0.001
Other heart disease	0.92 (0.16, 1.68)	2.44	0.019
Total CVD	1.67 (0.77, 2.58)	3.73	< 0.001
Sex			
Female	Ref.		
Male	1.01 (0.57, 1.43)	4.70	< 0.001
Country income classification^e^			
HIC	Ref.		
MIC	1.20 (0.10, 2.30)	2.19	0.033
Age group			
0 – 70 years	Ref.		
20 – 64 years	1.41 (0.86, 1.96)	5.15	< 0.001
30 – 74 years	2.14 (1.22, 3.06)	4.69	< 0.001

Meta-regression model was applied using a backward method for variable selection. The Hartung-Knapp adjustment, which accounts for the heterogeneity among studies, was applied. Adjusted covariates included CVD types, sex, country income classification, and age group. No multicollinearity or interaction was present. Model fitness was checked based on the corrected Akaike Information Criterion (AICc). The reduced model (AICc = 161.38) showed better fit compared to the full model, which included study time (AICc = 166.72)

### Publication’s bias

**Fig. 5:**
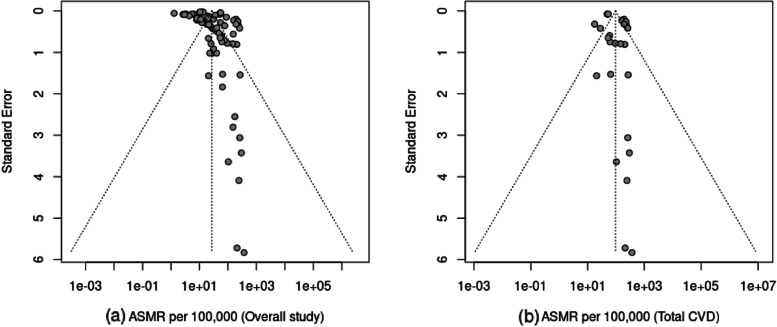
Funnel plot to assess publication bias of overall studies (**a**) and total CVD (**b**). (Note: Values on the x-axis refer to ASMR per 100,000 where 1e-01 = 0.1, 1e + 00 = 1, 1e + 01 = 10, 1e + 02 = 100 and 1e + 03 = 1000 and 1e + 05 = 10,000)

To assess publication bias in the included studies, we used funnel plots, Egger's test, and Begg's test. The funnel plot for all studies showed an asymmetrical distribution (Fig. 5a), indicating the possibility of publication bias or other sources of small-study effects. However, the Begg's and Egger's tests did not reach significance (p = 0.764 and p = 0.088, respectively), suggesting no evidence of publication bias in the meta-analysis. We also examined the funnel plot for studies that reported total CVD (ICD-10: I00-I99 or ICD-9: 350–459), which exhibited an almost symmetrical distribution (Fig. 5b). Furthermore, the Egger's and Begg's tests for total CVD were not significant (p = 0.559 and p = 0.084, respectively), indicating no presence of publication bias for total CVD. Therefore, based on our comprehensive assessment, we found no strong evidence of publication bias in our meta-analysis.

## Discussion

Premature mortality from CVD has significant socio-economic consequences and its ASMR varies widely across countries and regions, making it essential to understand the global burden of this disease. This meta-analysis and meta regression model combining the results of 15 studies demonstrated few key findings; (i) the pooled estimate of ASMR for premature CVD mortality was 27.0 per 100,000 populations where this estimate increased to 96.0 per 100,000 population when we included studies that only reported deaths from total CVD (ICD-10: I00-I99 or ICD-9: 350–459), (ii) specific CVD type for IHD higher ASMR than stroke, (iii) males had a higher ASMR than females, (iv) MICs had a higher ASMR than HICs, and (v) age group for 30–74 years had higher ASMR than other age threshold to defined premature mortality. Firstly, our analysis shows that the estimated ASMR for premature mortality related to total CVD (96.0 per 100,000 population) is comparable to the global estimate reported by Ji Zhang et al. [51]. The authors reported a global ASMR of 82.9 per 100,000 population for premature mortality from CVD in 2016 using data from the WHO's Global Health Estimates program. Therefore, our analysis provides further evidence to support the global estimation of premature CVD mortality and underscores the need for continued efforts to prevent and manage CVD.

Subgroup analysis by type of CVD and sex revealed significant disparities in the ASMRs. The ASMR for IHD was higher than that for stroke, consistent with the GBD 2019 study [4], which identified IHD as the leading cause of premature CVD mortality worldwide (accounted for approximately 7.8 million deaths in individuals under 70 years of age) and stroke was the second leading cause (accounted for approximately 3.6 million premature deaths). These findings accentuate the emphasis for targeted public health actions to intervene IHD and stroke, which remain major causes of premature mortality globally. Furthermore, the sex disparity in ASMRs align with a prior study employing global data estimated from the GBD and the WHO, in which male individuals had higher rates of premature CVD mortality than their female counterparts [4, 51, 52]. Further exploration of sex-specific differences in ASMRs by CVD type showed that males had a significantly higher ASMR for IHD and stroke than females. Over the past few decades, there has been increasing recognition of sex differences in the presentation, treatment, and outcomes of CVD. Studies have also demonstrated the variations in the effectiveness of risk factor control between sexes [53], propounding future research to explore the extent and underlying factors contributing to these disparities.

The observed disparities in ASMRs across different country income levels align with prior research indicating a greater burden of CVD and other NCDs in MICs [2, 54]. These disparities may stem from limited healthcare resources and restricted access to preventive interventions in MICs [55, 56]. Additionally, variations in the distribution of risk factors within these country settings contribute to the observed differences in mortality rates [57]. For instance, Sub-Saharan African countries exhibit a high prevalence of elevated blood pressure, while the Southeast Asia, East Asia, and Oceania region experiences elevated sodium consumption and diabetes rates. Additionally, South Asian countries face significant levels of ambient air pollution, which is a recognized risk factor for CVD mortality [57]. Moving forward, it is imperative for global health stakeholders and financiers to strategically examine ways to alleviate the burden of premature CVD mortality. This can be achieved by prioritizing healthcare resources and implementing targeted interventions in middle-income countries. By doing so, we can work towards reducing the impact of CVD and improving health outcomes in these regions.

Additionally, although study time is not significant in our meta regression model, the subgroup analysis revealed a significant increase in ASMR for premature CVD mortality between the periods 1990–1999 and 2000–2009, followed by a significant decrease in the period 2010–2019. While the reasons for this pattern are not entirely clear, it is possible that changes in behavioural lifestyle risk factors such as smoking, diet, and physical inactivity, as well as improvements in medical treatment and management of CVD, may have contributed to this trend. Over and beyond, our analysis of subgroups by income level uncovered a noteworthy reduction in ASMR from 2000–2009 to 2010–2019, for both MICs and HICs. According to the GBD study, there has been a global decline in premature CVD mortality rates over the past few decades with the greatest decline observed in HICs [2, 58]. These findings suggest that efforts to improve the prevention and management of CVD have been successful in reducing premature mortality in recent years, particularly in HICs. However, the burden of premature CVD mortality remains high in MICs, urging the need to ensure universal access to timely and affordable treatment for people living with CVD in these countries.

Addressing heterogeneity is a common challenge in meta-analyses, and our study employed sensitivity analyses to ensure the robustness of the results and enhance confidence in the observed disparities in ASMR estimates. These sensitivity analyses encompassed two crucial aspects: the removal of outlier studies and subgroup analysis based on different age thresholds. The exclusion of outlier studies significantly improved the precision of effect estimates and reduced heterogeneity, thereby increasing confidence in the observed disparities in ASMRs, particularly for CVD type (total CVD). Notably, when one selected study utilizing multi-country data from three distinct regions in the USA (North America, Latin America, and the non-Latin Caribbean) was excluded, the estimated ASMR for total CVD substantially decreased from 96.04 to 46.69. This study reported the highest ASMR for premature total CVD mortality in the non-Latin Caribbean region. Consequently, utilizing the estimated ASMR for total CVD mortality (96.04) without removing the outlier allows for a more generalized and comparable approach to the findings of a global study on premature total CVD mortality (82.9 per 100,000) [51]. On the other hand, subgroup analyses demonstrated consistent patterns both before and after removing outliers, indicating the robustness of the findings and their limited dependence on outlier data for most subgroup analyses. Additionally, the sensitivity analysis based on different age thresholds provided a comprehensive exploration of the impact of age on premature CVD mortality. While the estimated ASMR varied across age groups compared to the original selection of 15 studies, the patterns remained consistent. For instance, the ASMR for premature CVD mortality among males remained higher than females, and the estimated ASMR for MICs remained higher than HICs.

The present meta-analysis highlights several recommendations to address premature CVD mortality. Firstly, it is crucial to reinforce prevention strategies that focus on promoting healthy lifestyles, managing risk factors, and implementing measures to prevent CVD. This includes encouraging regular physical activity, a balanced diet, and avoiding tobacco use, as well as managing hypertension and diabetes effectively. Secondly, tailoring prevention strategies to specific populations, considering factors like sex and CVD types, is suggested. Targeted interventions and educational campaigns should address specific risk factors and barriers to healthcare access faced by different populations. Improving healthcare services' accessibility, particularly in MICs, is vital for preventing premature CVD mortality. Thirdly, monitoring trends in ASMR over time will help evaluate the effectiveness of prevention strategies and identify areas requiring more resources. Fourthly, more research is needed to understand the complex factors contributing to premature CVD mortality, in particular structural determinants or social determinants of health inequities. Standardizing data collection and reporting of mortality rates by incorporating standardized ICD codes and providing comprehensive information on population characteristics will facilitate comparability across studies and identify potential disparities. Adopting a standardized tool for assessing the quality of included studies, such as the NOS or other established quality assessment tools, is also recommended to enhance the reliability and comparability of the findings and support evidence-based interventions and policies to address this significant public health issue.

### Strengths and limitations

This study has several strengths that contribute to its robustness and reliability. Firstly, it includes studies from both HICs and MICs, providing a comprehensive and up-to-date estimate of the global prevalence of premature CVD mortality. Additionally, the study employed standardized study rating instruments and adhered to relevant guidelines for systematic reviews and meta-analyses, ensuring the rigor and validity of the research. The review closely examined the materials and methods of all included papers during the screening stage to assess sample representativeness, following recommended steps [59]. When authors do not indicate any deviations from representing the population, the samples used in their studies are assumed to be representative, aligning with standard practices for meta-analyses.

However, there are important limitations that should be acknowledged when interpreting the results. One of our assumptions is that the data sources in individual studies are representative of the national population. Although representativeness was evaluated in the quality assessment, we can’t completely rule out potential selection bias. Obtaining representative publications from developing countries, particularly LMICs, is challenging due to research barriers [60]. We used a comprehensive search strategy, multiple databases, and cross-referencing to allow identification of a wide range of publications. Nevertheless, we identified limited publications in certain regions, particularly LMICs, which affects the generalizability of our results. Furthermore, high heterogeneity among the included studies may impact the overall quality of evidence, as variations in characteristics could introduce biases. Subgroup analyses were conducted to explore sources of heterogeneity, but these methods have limitations and require cautious interpretation.

## Conclusions

In conclusion, our review and meta-analysis of 15 studies provides estimates of the global age-standardized mortality rate for premature CVD mortality. The overall ASMR estimate for premature CVD mortality from all studies was 27.0 per 100,000 population. However, when specifically considering studies that reported deaths from total CVD, the estimate increased to 96.0 per 100,000 population, indicating substantial heterogeneity among the included studies. Notably, our meta-regression model demonstrated significant variations in ASMRs based on CVD type, sex, income country level, and age threshold used to define premature mortality. Specifically, we observed that IHD exhibited the highest ASMR compared to stroke, indicating the differential impact of various CVD subtypes on premature mortality. Furthermore, males experienced a higher ASMR compared to females, highlighting sex disparities in CVD-related mortality. Additionally, MICs displayed higher ASMRs than HICs, suggesting the influence of socio-economic factors on premature CVD mortality. Furthermore, our analysis revealed that the age group of 30–74 years had a higher ASMR compared to the broader age range of 0–74 years, emphasizing the importance of targeted interventions for this specific age cohort. Overall, our findings provide crucial insights into the global patterns and disparities in premature CVD mortality. These findings have significant implications for public health strategies to reduce the burden of premature CVD mortality and improve global health outcomes.

### Supplementary Information

**Additional file 1.** 

## Data Availability

The datasets analysed during the current study are available from the corresponding author on reasonable request.

## Abbreviations

ASMR: Age-standardized mortality rate
CVD: Cardiovascular disease
GBD: Global Burden of Disease
IHD: Ischemic heart disease
CBVD: Cerebrovascular disease
LMICs: Low- and middle-income countries
HICs: High-income countries
MICs: Middle-income countries
NCD: Non-communicable diseases
SDG: Sustainable Development Goal
PROSPERO: Prospective Register of Systematic Reviews
PRISMA: Preferred Reporting Items for Systematic Reviews and Meta-analysis
WOS: Web of Science
CENTRAL: Cochrane Central Register of Controlled Trials
NOS: Newcastle–Ottawa Scale
ICD: International Classification of Disease
SE: Standard error
CI: Confidence interval
PI: Prediction interval
NMRR: National Medical Research Register
USM: Universiti Sains Malaysia
