# Supplementary material for: The global estimate of premature cardiovascular mortality: a systematic review and meta-analysis of age-standardized mortality rate
Source: BMC Public Health. 2023 Aug 16;23:1561. doi: 10.1186/s12889-023-16466-1 (PMC10429077; doi:10.1186/s12889-023-16466-1)
Supplement: Supplementary file 1 — Additional file 1. [file 12889_2023_16466_MOESM1_ESM.docx]

**Supplement 1**

**Table S1: Search Terms**

| Databases | Search terms |
| --- | --- |
| Web of Science | AB=("cardiovascular disease*" OR "CVD" OR "coronary disease*" OR "coronary heart disease*" OR "heart disease*" OR "cardiac disease*" OR "cardiac disorder*" OR "heart disorder*" OR "cardiac arrhythmia*" OR "cardiac dysrhythmia*" OR "atrial fibrillation*" OR "coronary artery disease*" OR "coronary arteriosclerosis" OR "Coronary Atherosclerosis" OR "myocardial ischemia*" OR "ischemic heart disease*" OR "myocardial infarction*" OR "cardiovascular stroke*" OR "heart attack*" OR "cardiogenic shock" OR "acute coronary syndrome*" OR "angina pectori*" OR "Cerebrovascular Disorders" OR "Cerebrovascular Disorders" OR "intracranial vascular disease*" OR "intracranial vascular disorder*" OR "cerebrovascular disease*" OR "brain vascular disorder*" OR "cerebrovascular occlusion*" OR "cerebrovascular insufficiency*" OR "brain ischemia" OR "carotid artery disease*" OR "cerebral vessel diseases" OR "intracranial arterial diseases" OR "intracranial hemorrhage*"OR "cerebral hemorrhage*" OR "stroke*" OR "cerebrovascular accident*" OR "CVA" OR "brain vascular accident*" OR "intracranial arteriosclerosis" OR "cerebral arteriosclerosis" OR "cerebral atherosclerosis" )  AND  TI=("premature mortality" OR "premature death" OR “age standardized mortality rate” OR “standardized death rate” OR “ASMR” OR "years of life lost" OR "life expectancy " )  AND  Articles (Document Types) and English (Languages) and Review Articles (Exclude – Document Types) and Animals or Mice (Exclude – MeSH Headings) |
| Pubmed | Search: cardiovascular disease[MeSH Terms] OR "cardiovascular disease*"[Title/Abstract] OR "CVD"[Title/Abstract] OR "coronary disease*"[Title/Abstract] OR "coronary heart disease*"[Title/Abstract] OR "heart disease*"[Title/Abstract] OR "cardiac disease*"[Title/Abstract] OR "cardiac disorder*"[Title/Abstract] OR "heart disorder*"[Title/Abstract] OR "cardiac arrhythmia*"[Title/Abstract] OR "cardiac dysrhythmia*"[Title/Abstract] OR "atrial fibrillation*"[Title/Abstract] OR "coronary artery disease*"[Title/Abstract] OR "coronary arteriosclerosis"[Title/Abstract] OR "Coronary Atherosclerosis"[Title/Abstract] OR "myocardial ischemia*"[Title/Abstract] OR "ischemic heart disease*"[Title/Abstract] OR "myocardial infarction*"[Title/Abstract] OR "cardiovascular stroke*"[Title/Abstract] OR "heart attack*"[Title/Abstract] OR "cardiogenic shock"[Title/Abstract] OR "acute coronary syndrome*"[Title/Abstract] OR "angina pectori*"[Title/Abstract] OR "Cerebrovascular Disorders"[Title/Abstract] OR "intracranial vascular disease*"[Title/Abstract] OR "intracranial vascular disorder*"[Title/Abstract] OR "cerebrovascular disease*"[Title/Abstract] OR "brain vascular disorder*"[Title/Abstract] OR "cerebrovascular occlusion*"[Title/Abstract] OR "cerebrovascular insufficiency*"[Title/Abstract] OR "brain ischemia"[Title/Abstract] OR "carotid artery disease*"[Title/Abstract] OR "cerebral vessel diseases"[Title/Abstract] OR "intracranial arterial diseases"[Title/Abstract] OR "intracranial hemorrhage*"OR "cerebral hemorrhage*"[Title/Abstract] OR "stroke*"[Title/Abstract] OR "cerebrovascular accident*"[Title/Abstract] OR "CVA"[Title/Abstract] OR "brain vascular accident*"[Title/Abstract] OR "intracranial arteriosclerosis"[Title/Abstract] OR "cerebral arteriosclerosis"[Title/Abstract] OR "cerebral atherosclerosis"[Title/Abstract]  AND  Search: "premature mortality"[Title/Abstract] OR "premature death"[Title/Abstract] OR "years of life lost"[Title/Abstract] "age standardized mortality rate"[Title/Abstract] OR "standardized death rate*"[Title/Abstract] OR "Life Expectancy"[Title/Abstract]  AND  Limit to English and limit to Human |
| Scopus | TITLE-ABS-KEY ( "cardiovascular disease*" OR "CVD" OR "coronary disease*" OR "coronary heart disease*" OR "heart disease*" OR "cardiac disease*" OR "cardiac disorder*" OR "heart disorder*" OR "cardiac arrhythmia*" OR "cardiac dysrhythmia*" OR "atrial fibrillation*" OR "coronary artery disease*" OR "coronary arteriosclerosis" OR "Coronary Atherosclerosis" OR "myocardial ischemia*" OR "ischemic heart disease*" OR "myocardial infarction*" OR "cardiovascular stroke*" OR "heart attack*" OR "cardiogenic shock" OR "acute coronary syndrome*" OR "angina pectori*" OR "Cerebrovascular Disorders" OR "Cerebrovascular Disorders" OR "intracranial vascular disease*" OR "intracranial vascular disorder*" OR "cerebrovascular disease*" OR "brain vascular disorder*" OR "cerebrovascular occlusion*" OR "cerebrovascular insufficiency*" OR "brain ischemia" OR "carotid artery disease*" OR "cerebral vessel diseases" OR "intracranial arterial diseases" OR "intracranial hemorrhage*" OR "cerebral hemorrhage*" OR "stroke*" OR "cerebrovascular accident*" OR "CVA" OR "brain vascular accident*" OR "intracranial arteriosclerosis" OR "cerebral arteriosclerosis" OR "cerebral atherosclerosis" )  AND  TITLE ("premature mortality" OR "premature death" OR “age standardized mortality rate” OR “ASMR” OR “standardized death rate” OR "years of life lost" OR "life expectancy" )  AND  ( LIMIT-TO ( DOCTYPE , "ar" ) ) AND ( LIMIT-TO ( LANGUAGE , "english" ) ) |
| Cochrane Central Register of Controlled Trials (CENTRAL) | MeSH descriptor: [Cardiovascular Diseases] explode all trees  AND  "premature mortality" OR "premature death" OR "years of life lost" OR " age standardized mortality rate " OR "ASMR" OR “standardized death rate” OR "life expectancy" |

**Supplement 2**

**Newcastle-Ottawa Scale adapted for quality assessment of cross-sectional study for the systematic review “The global estimate of premature cardiovascular mortality: a systematic review and meta-analysis of age standardized mortality rate”**

The Newcastle-Ottawa Scale (NOS) is a tool used for assessing the quality of non-randomized studies in meta-analyses [1]. The NOS uses a star rating system to evaluate the quality of individual studies based on three categories: selection of study groups, comparability of study groups, and ascertainment of outcomes. The original version of NOS is for cohort and cohort study [1]. Herzog et al., [2] created the adapted version of NOS for cross-sectional studies. We modified the adapted version of the NOS by Herzog et al., [2] to suit our systematic review (Table 1).

Table 1: The adapted version of Newcastle-Ottawa Scale for this study

| Components | Newcastle-Ottawa Scale adapted for cross-sectional studies |
| --- | --- |
| Selection | 1) Representativeness of the sample:  a) Truly representative of the average in the target population. *  (use death registry database or vital registration represent state or country level mortality data for CVD death)  b) Somewhat representative of the average in the target population. *  (State or country level mortality data but select specific CVD type or subgroup population)  c) Selected group of users.  d) No description of the sampling strategy.  2) Sample size:  a) Justified and satisfactory. * (reported number of death or completion of mortality database)  b) Not justified.  3) Non-respondents:  a) Comparability between respondents and non-respondents’ characteristics is established, and the response rate is satisfactory. * (reported comparable population as the denominator for the ASMR calculation)  b) The response rate is unsatisfactory, or the comparability between respondents and non-respondents is unsatisfactory.  c) No description of the response rate or the characteristics of the responders and the non-responders.  4) Ascertainment of the exposure (or outcome):  a) Validated measurement tool. ** (defined premature mortality with age range)  b) Non-validated measurement tool, but the tool is available or described.* (reported premature mortality without a specified age range)  c) No description of the measurement tool.  (Maximum 5 stars) |
| Comparability | 1) The subjects in different outcome groups are comparable, based on the study design or analysis. Confounding factors are controlled.  a) The study controls for the most important factor (using standard population for age adjusted mortality rate). *  b) The study control for any additional factor. * (adjusted by sex)  (Maximum 2 stars) |
| Outcome | 1) Assessment of the outcome:  a) Independent blind assessment. **  b) Record linkage. ** (use death registry data source and classified cause of death based on International Classification of Disease, ICD code)  c) Self report. *  d) No description.  2) Statistical test:  a) The statistical test used to analyze the data is clearly described and appropriate, and the measurement of the association is presented, including confidence intervals and the probability level (p value). * (report clear step of calculating ASMR)  b) The statistical test is not appropriate, not described or incomplete.  (Maximum 3 stars) |
| Scoring | Very Good Studies: 9-10 points  Good Studies: 7-8 points  Satisfactory Studies: 5-6 points  Unsatisfactory Studies: 0 to 4 points |

References

1. Wells GA, Shea B, O’Connell D, Peterson J, Welch V, Losos M, et al. The Newcastle-Ottawa Scale (NOS) for assessing the quality of nonrandomised studies in meta-analyses. Oxford; 2000.
2. Herzog R, Álvarez-Pasquin Maand Díaz C, del Barrio JL, Estrada JM, Gil Á. Are healthcare workers’ intentions to vaccinate related to their knowledge, beliefs and attitudes? A systematic review. BMC Public Health. 2013;13: 1–17.

Based on criteria from the Newcastle-Ottawa Scale adapted for cross-sectional studies, we assess the quality of each of the 15 selected studies in our systematic review, which is presented in Table 2.

Table 2: Quality assessment of 15 selected studies using an adapted version of the Newcastle-Ottawa Scale for cross-sectional studies

| Component | | Selected studies | | | | | | | | | | | | | | |
| --- | --- | --- | --- | --- | --- | --- | --- | --- | --- | --- | --- | --- | --- | --- | --- | --- |
|  |  | ^1^ | ^2^ | ^3^ | ^4^ | ^5^ | ^6^ | ^7^ | ^8^ | ^9^ | ^10^ | ^11^ | ^12^ | ^13^ | ^14^ | ^15^ |
| Selection | |  |  |  |  |  |  |  |  |  |  |  |  |  |  |  |
|  | Representativeness of the sample (use death registry/vital registration data) | * | * | * | * | * | * | * | * | * | * | * | * | * | * | * |
|  | Sample Size (reported number of death) |  | * |  | * |  | * | * |  | * |  | * | * | * | * | * |
|  | None- respondents (reported comparable population as the denominator for the ASMR calculation) | * | * | * | * | * | * | * | * | * |  | * | * | * |  | * |
|  | Ascertainment of exposure or outcome (defined premature mortality with age range) | * * | * | * * | * * | * * | * * | * * | * * | * * | * * | * * | * * | * * | * * | * * |
| Comparability | |  |  |  |  |  |  |  |  |  |  |  |  |  |  |  |
|  | Controls for the most important factor (using standard population for age adjusted mortality rate) | * | * | * | * | * | * | * | * | * | NR | * | * | * | * | * |
|  | Control for any additional factor (adjusted by sex) | * | * |  | * | * | * | * | * |  |  | * | * | * | * | * |
| Outcome | |  |  |  |  |  |  |  |  |  |  |  |  |  |  |  |
|  | Assessment of the outcome (use ICD code to classified cause of death) | *  * | *  * | *  * | *  * | *  * | *  * | *  * | *  * | *  * | *  * | *  * | *  * | *  * | * * | * * |
|  | Statistical test (report clear step of calculating ASMR) | * | * | * | * | * | * | * | * | * |  | * | * | * | * | * |
| Total Score | | 9 | 9 | 8 | 10 | 9 | 10 | 10 | 9 | 9 | 5 | 10 | 10 | 10 | 9 | 10 |
|  | | V G | V G | G | V G | V G | V G | V G | V G | V G | S | V G | V G | V G | V G | V G |

*ASMR: age standardized mortality rate. NR: not reported. VG: very good. G: good. S: satisfactory.

Selected studies

1. Dani SS, Lone AN, Javed Z, Khan MS, Khan MZ, Kaluski E, et al. Trends in Premature Mortality From Acute Myocardial Infarction in the United States, 1999 to 2019. J Am Heart Assoc. 2022 Jan;11(1).

2. Santric Milicevic M, Bjegovic V, Terzic Z, Vukovic D, Kocev N, Marinkovic J, et al. Serbia within the European context: An analysis of premature mortality. Popul Health Metr. 2009;7(1):1–10.

3. Yang H, Fu Y, Hong X, Yu H, Wang W, Sun F, et al. `Trend in premature mortality from four major NCDs in Nanjing, China, 2007-2018’. BMC Public Health. 2021 Nov;21(1).

4. Istilli PT, de Souza Teixeira CR, Zanetti ML, Dias Lima RA, Alves Pereira MC, Ricci WZ. Assessment of premature mortality for noncommunicable diseases. Rev Bras Enferm. 2020;73(2).

5. Puska P, Vartiainen E, Tuomilehto J, Salomaa V, Nissinen A. Changes in premature deaths in Finland: successful long-term prevention of cardiovascular diseases. Bull World Health Organ. 1998;76(4):419–25.

6. Moryson W, Stawinska-Witoszynska B. TRENDS IN PREMATURE MORTALITY RATES AMONGTHE POLISH POPULATION DUE TO CARDIOVASCULAR DISEASES. Int J Occup Med Environ Health. 2022;35(1):27–38.

7. Hervella MI, Carratalá-Munuera C, Orozco-Beltrán D, López-Pineda A, Bertomeu-González V, Gil-Guillén VF, et al. Trends in premature mortality due to ischemic heart disease in Spain from 1998 to 2018. Revista Española de Cardiología (English Edition). 2021 Oct;74(10):838–45.

8. Best AF, Haozous EA, de Gonzalez AB, Chernyavskiy P, Freedman ND, Hartge P, et al. Premature mortality projections in the USA through 2030: a modelling study. LANCET PUBLIC HEALTH. 2018 Aug;3(8):E374–84.

9. Wijnen A, Bishop K, Joshy G, Zhang Y, Banks E, Paige E. Observed and predicted premature mortality in Australia due to non-communicable diseases: a population-based study examining progress towards the WHO 25X25 goal. BMC Med. 2022 Feb;20(1).

10. Pinlac PA V, Soonthornworasiri N. Descriptive and predictive time series analysis of premature mortality from noncommunicable disease among Filipinos. Acta Med Philipp [Internet]. 2016;50(3):144–51. Available from: https://www.scopus.com/inward/record.uri?eid=2-s2.0-85017631541&partnerID=40&md5=7db197d74ff0f52885aa7479f19e7052

11. MARIANI J, MONSALVO M, PRIETO AF, MACCHIA A. Premature death from stroke and socioeconomic status in Argentina. Rev Argent Cardiol. 2016;84(2):114–9.

12. Gómez-Mart\’\inez L, Orozco-Beltran D, Quesada JA, Bertomeu-González V, Gil-Guillen VF, Lopez-Pineda A, et al. Trends in premature mortality due to heart failure by autonomous community in Spain: 1999 to 2013. Revista Española de Cardiolog{\’\i}a (English Edition). 2018;71(7):531–7.

13. Song S, Ma G, Trisolini MG, Labresh KA, Smith SC, Jin Y, et al. Evaluation of Between-County Disparities in Premature Mortality Due to Stroke in the US. JAMA Netw Open. 2021 May;4(5).

14. Gawryszewski VP, Souza M de FM de. Mortality due to cardiovascular diseases in the Americas by region, 2000-2009. Sao Paulo Medical Journal. 2014;132:105–10.

15. Jin Y, Song S, Zhang L, Trisolini MG, Labresh KA, Smith Jr SC, et al. Disparities in premature cardiac death among US counties from 1999--2017: temporal trends and key drivers. J Am Heart Assoc. 2020;9(15):e016340.

**Supplement 3**


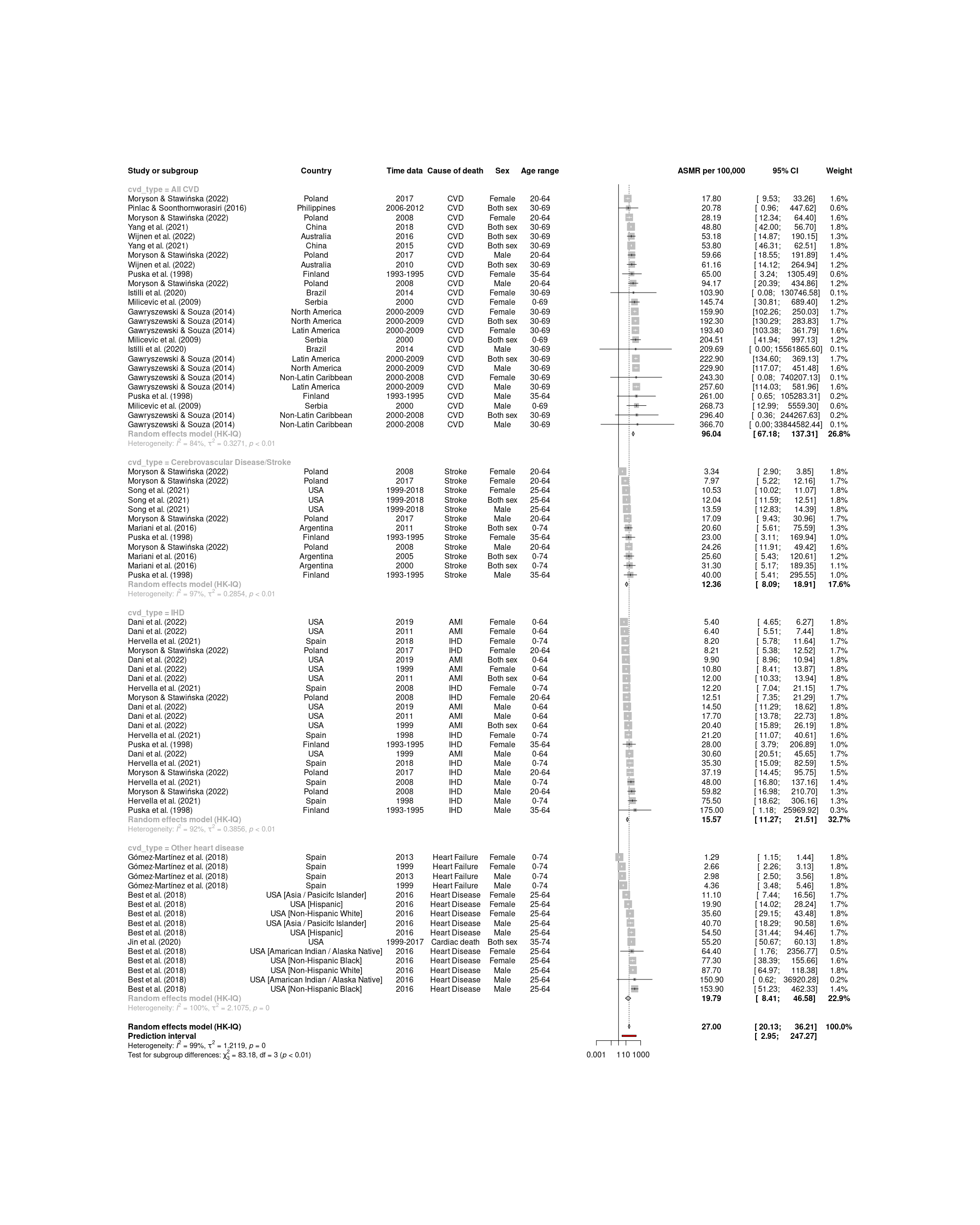


**Figure S1: Forest plot of ASMR per 100,000 population from premature CVD mortality (all studies) with subgroup analysis by CVD types**


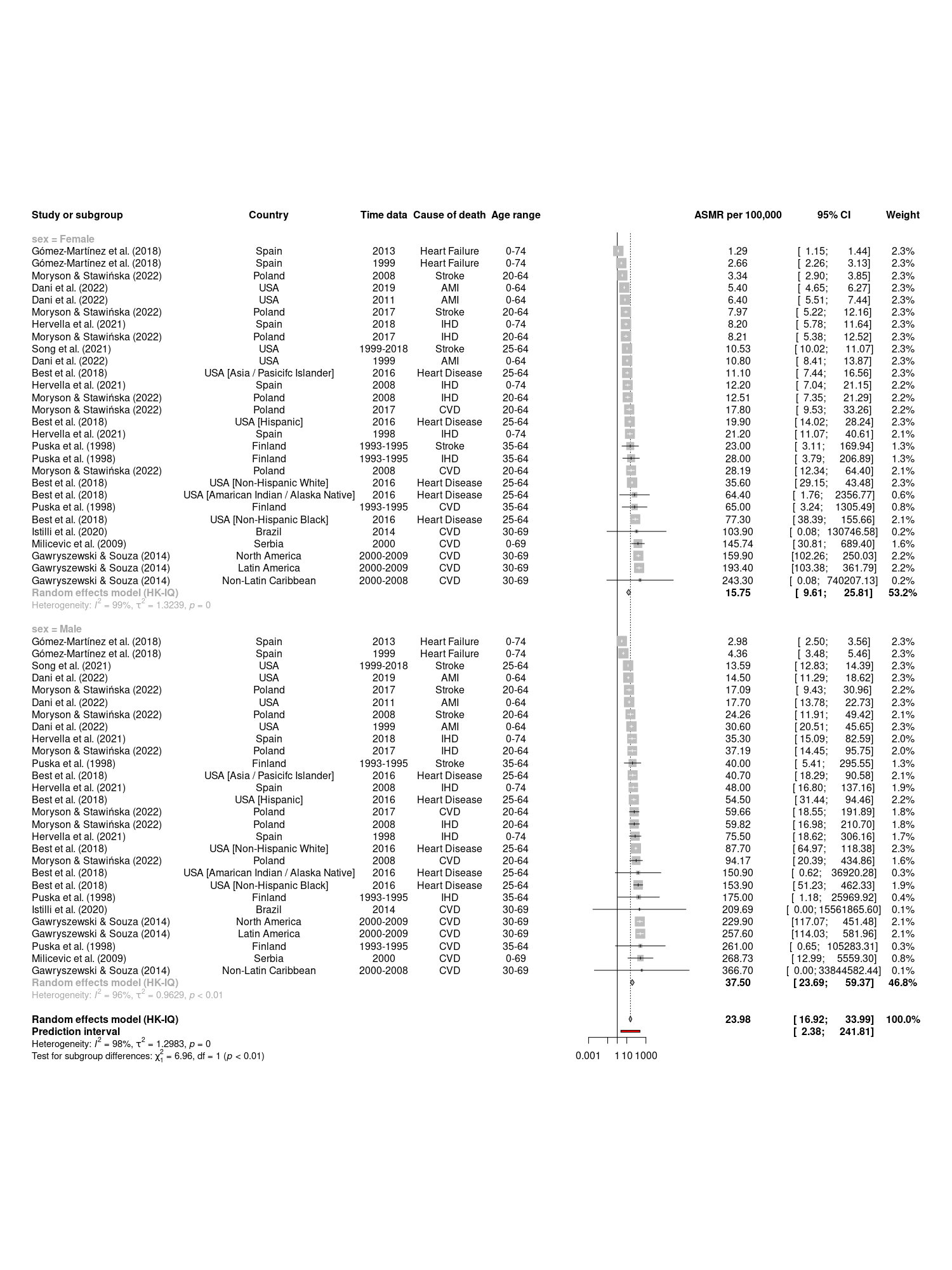


**Figure S2: Forest plot of ASMR per 100,000 population from premature CVD mortality (all studies) with subgroup analysis by sex (excluded both sex)**


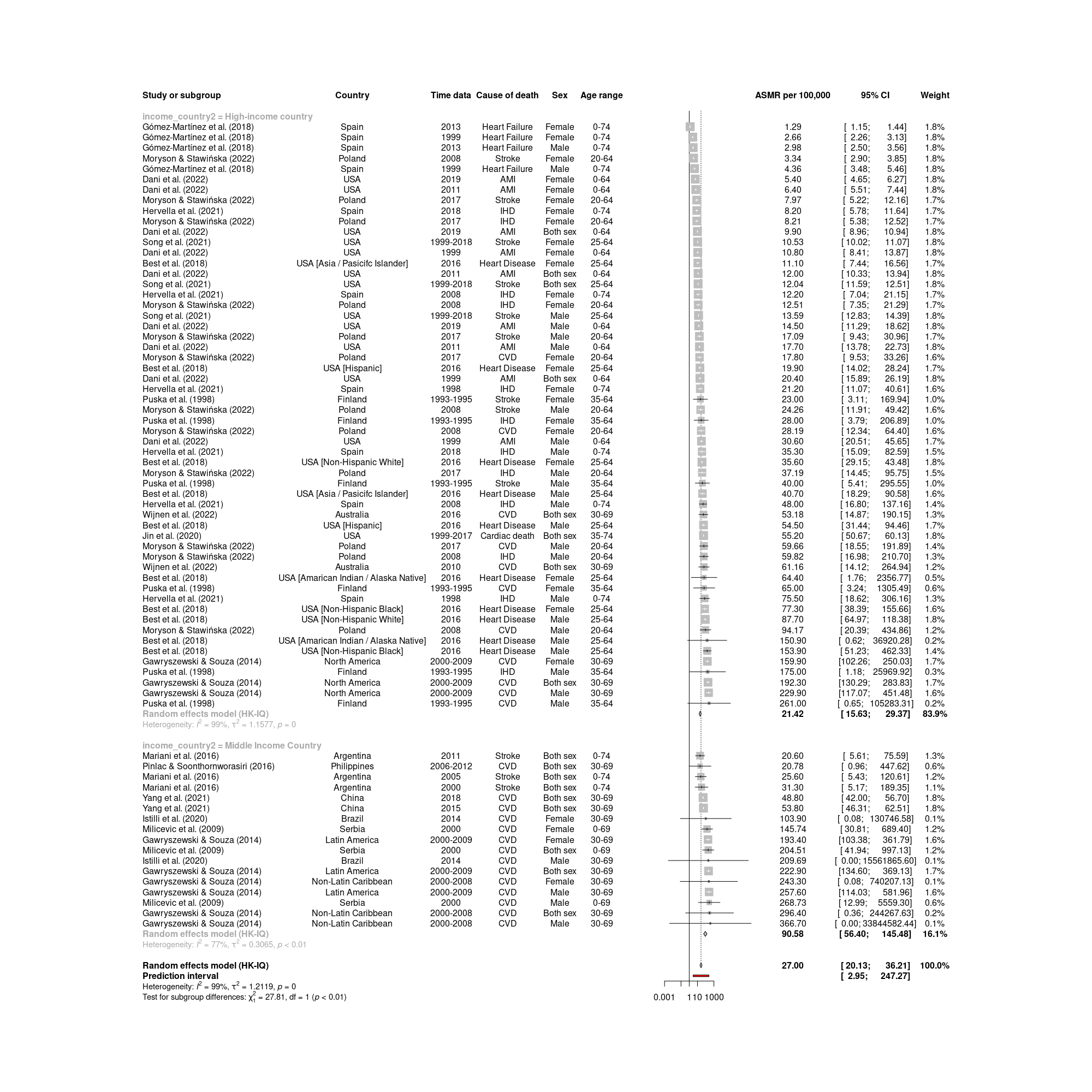


**Figure S3:** **Forest plot of ASMR per 100,000 population from premature CVD mortality (all studies) with subgroup analysis by income country level**


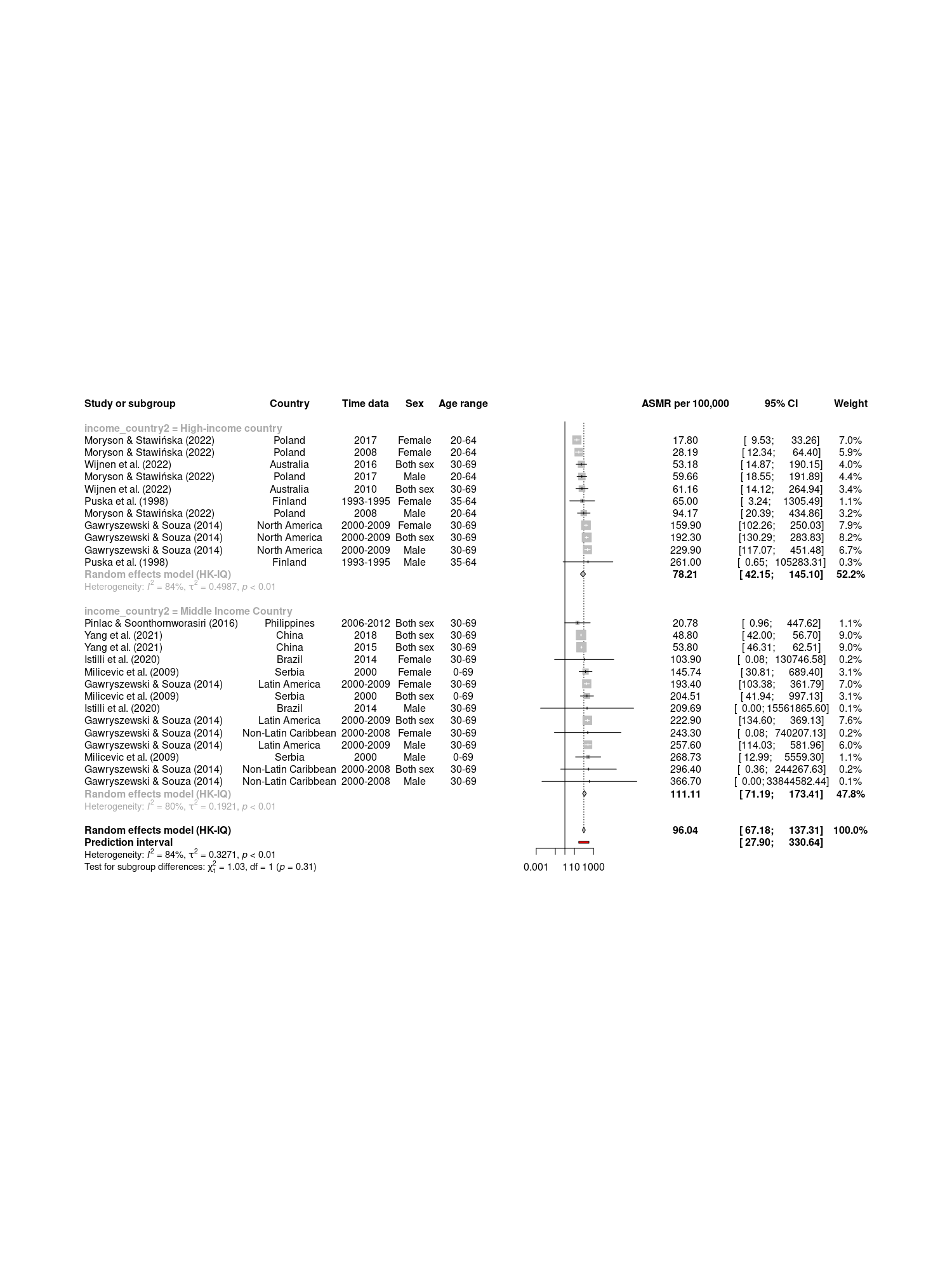


**Figure S4: Forest plot of ASMR per 100,000 population from premature mortality among total CVD** **(ICD-10 codes I00-I99 or ICD-9 codes 350-459) with subgroup analysis by income country classification**


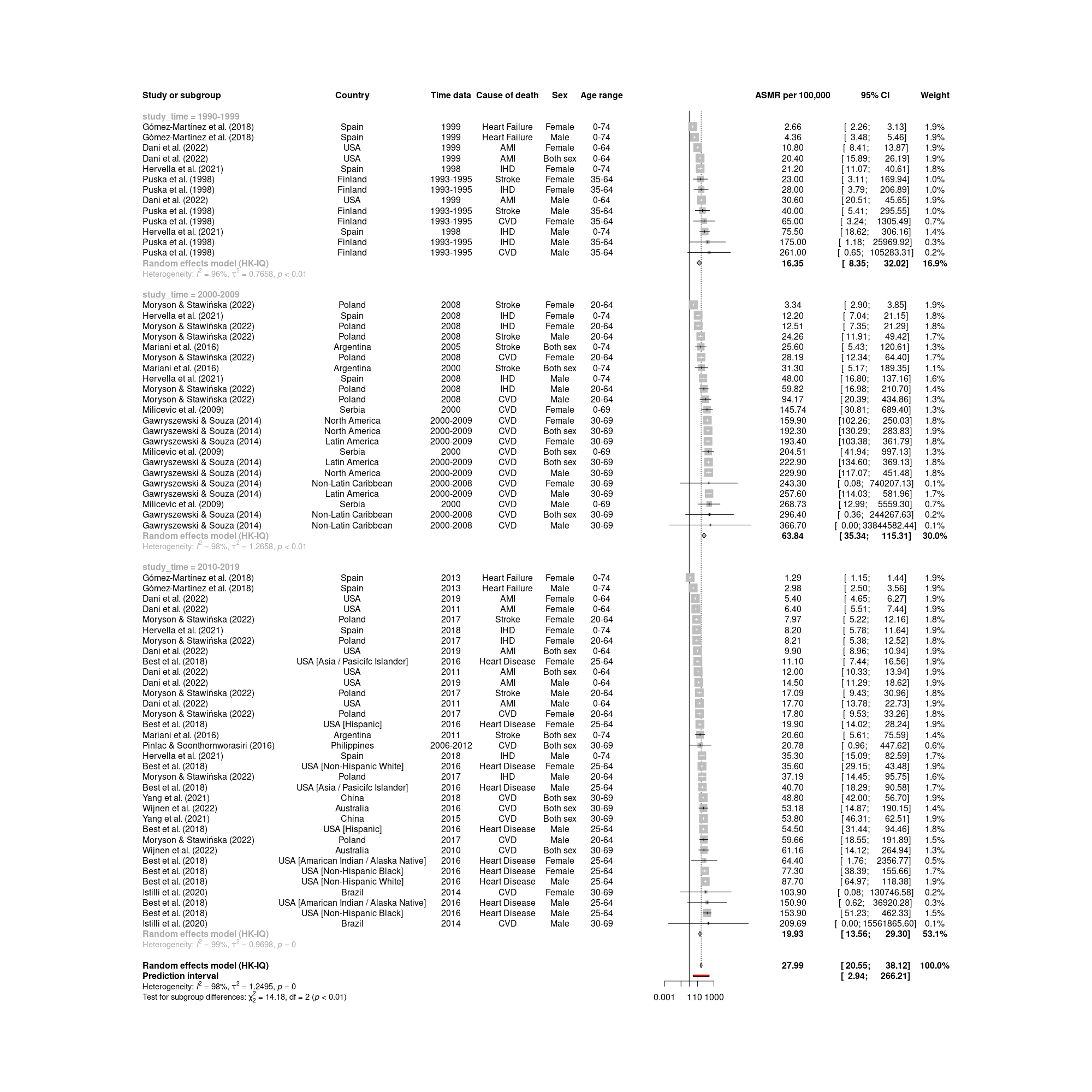


**Figure S5: Forest plot of ASMR per 100,000 population from premature CVD mortality (all studies) with subgroup analysis by study time**


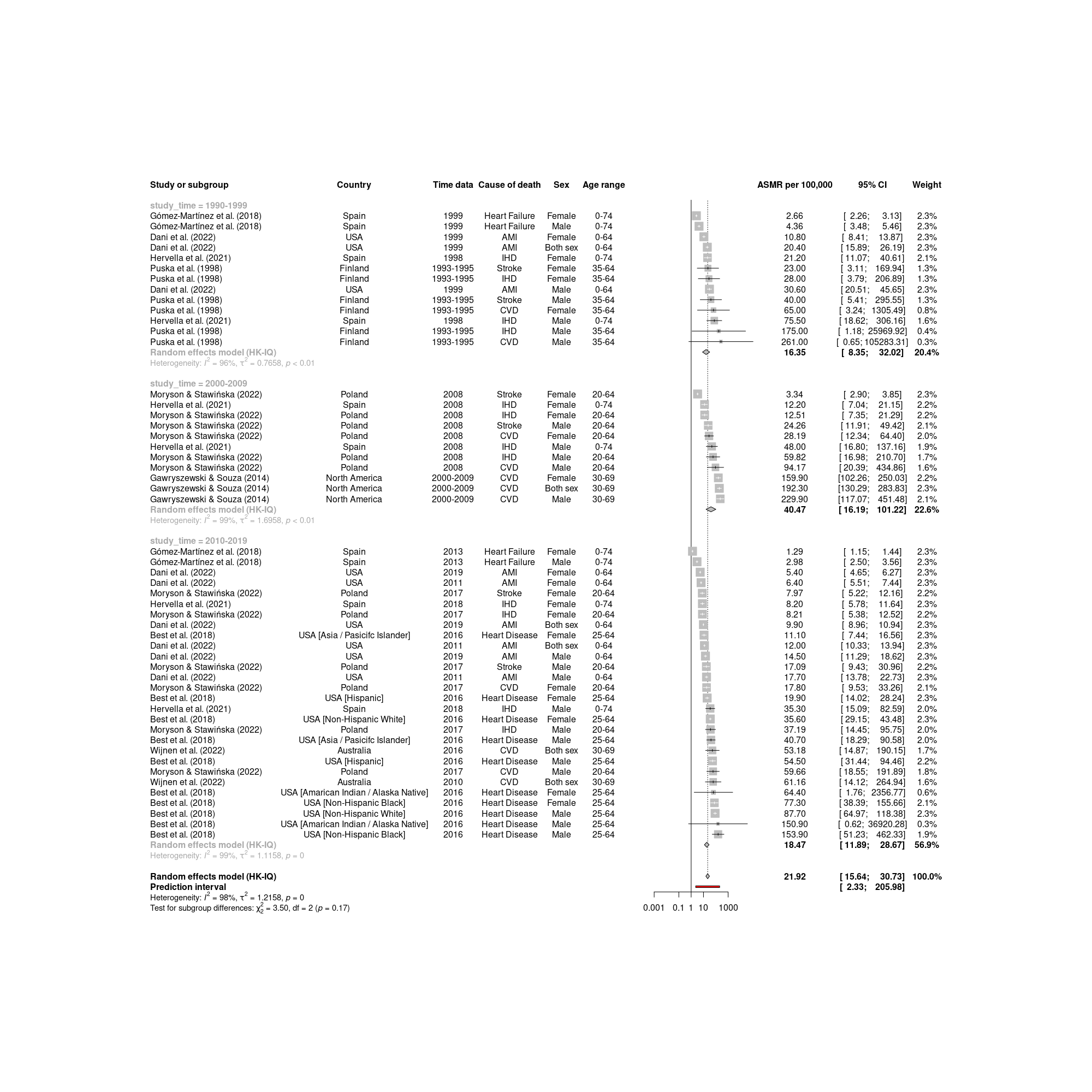


**Figure S6: Forest plot time-stratified analysis of premature CVD mortality (ASMR per 100,000 population) among high income countries**


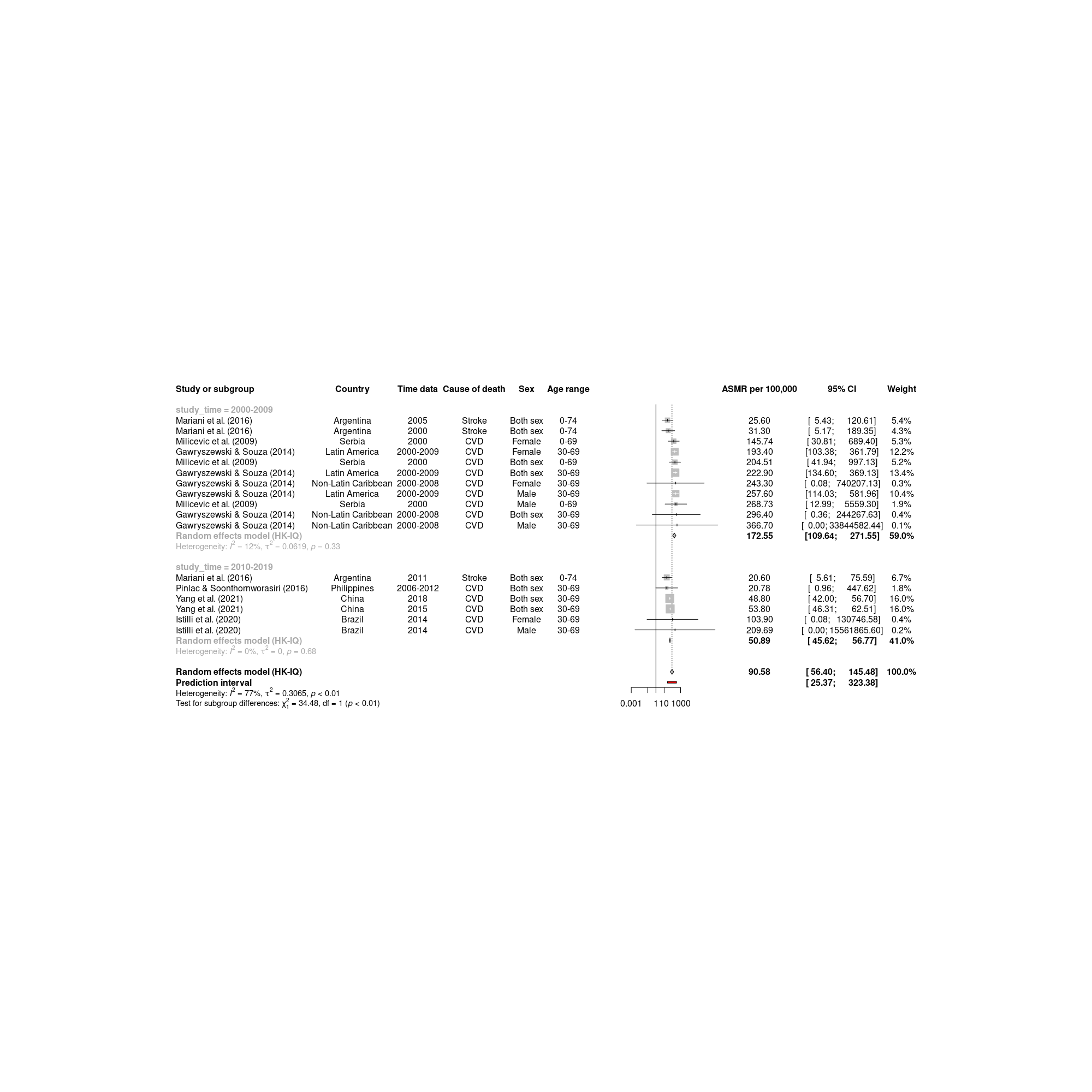


**Figure S7: Forest plot time-stratified analysis of premature CVD mortality (ASMR per 100,000 population) among middle (low and upper) income countries**
